# Morphological connectivity differences in Alzheimer's disease correlate with gene transcription and cell‐type

**DOI:** 10.1002/hbm.26512

**Published:** 2023-10-17

**Authors:** Huiying Yu, Yanhui Ding, Yongbin Wei, Martin Dyrba, Dong Wang, Xiaopeng Kang, Weizhi Xu, Kun Zhao, Yong Liu, for the Alzheimer's Disease Neuroimaging Initiative

**Affiliations:** ^1^ School of Information Science and Engineering Shandong Normal University Jinan China; ^2^ School of Artificial Intelligence Beijing University of Posts and Telecommunications Beijing China; ^3^ German Center for Neurodegenerative Diseases (DZNE) Rostock Germany; ^4^ School of Artificial Intelligence University of Chinese Academy of Sciences Beijing China

**Keywords:** Alzheimer's disease, cell‐type, gene expression, regional radiomics similarity network, structural connectomes

## Abstract

Alzheimer's disease (AD) is one of the most prevalent forms of dementia in older individuals. Convergent evidence suggests structural connectome abnormalities in specific brain regions are linked to AD progression. The biological basis underpinnings of these connectome changes, however, have remained elusive. We utilized an individual regional mean connectivity strength (RMCS) derived from a regional radiomics similarity network to capture altered morphological connectivity in 1654 participants (605 normal controls, 766 mild cognitive impairment [MCI], and 283 AD). Then, we also explored the biological basis behind these morphological changes through gene enrichment analysis and cell‐specific analysis. We found that RMCS probes of the hippocampus and medial temporal lobe were significantly altered in AD and MCI, with these differences being spatially related to the expression of AD‐risk genes. In addition, gene enrichment analysis revealed that the modulation of chemical synaptic transmission is the most relevant biological process associated with the altered RMCS in AD. Notably, neuronal cells were found to be the most pertinent cells in the altered RMCS. Our findings shed light on understanding the biological basis of structural connectome changes in AD, which may ultimately lead to more effective diagnostic and therapeutic strategies for this devastating disease.

## Graphical abstract

Yu et al., examined morphological connectivity differences in Alzheimer's disease using the R2SN approach. Those findings link these differences to clinical measures, high‐risk AD genes, and specific cellular processes, shedding light on the biological basis of structural connectome changes in AD.

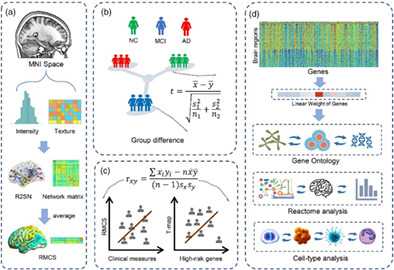

## 1 INTRODUCTION

Alzheimer's disease (AD) is one of the most common type of dementia, which is manifested as the decline of memory, language ability, and cognition (Goedert & Spillantini, 2006). The atrophy in morphological brain regions is one of the most common hallmarks of AD, especially in the hippocampus and medial temporal lobe (Dickerson et al., 2009; Plachti et al., 2020; Štěpán‐Buksakowska et al., 2014). However, traditional voxel‐based morphometry cannot comprehensively reflect the altered morphometry in AD. The intricate workings of the brain facilitate the transformation of information, making it a complex system of paramount importance (Bullmore & Sporns, 2012). Therefore, employing a large‐scale network founded upon inter‐regional morphological similarity is imperative in understanding brain organization instead of isolated analysis of specific brain regions (Alexander‐Bloch et al., 2013; Tijms et al., 2012; Zhao et al., 2021).

The employment of interregional similarity networks, particularly in the context of structural covariance networks (SCNs), has demonstrated the capacity to capture synergistic alterations in morphological architecture across distinct brain regions (Bethlehem et al., 2017; Binnewijzend et al., 2014; Dai et al., 2019; Kim et al., 2016; Li et al., 2021; Yao et al., 2010; Yu et al., 2018; Zheng et al., 2015). Furthermore, SCNs have exhibited efficacy in investigating the dysfunction of the connectome within the spectrum of AD, thereby yielding a comprehensive array of anatomical indices that serve to discriminate AD and demarcate subtypes of MCI patients (Fu et al., 2021; Montembeault, Rouleau, Provost, Brambati, & Alzheimer's Disease Neuroimaging, 2016; Tijms et al., 2012; Yu et al., 2018; Zhao et al., 2021; Zhao et al., 2022; Zhao et al., 2023). While the SCN has garnered diverse applications in the context of AD, how the connectome changes in AD and what is related to those variations are not well‐established. Neuroimaging genomics has introduced novel insights into the association between disease‐specific alterations and microscale architectural changes or genetic predisposition. This advancement has found application in significant psychiatric conditions such as major depressive disorder and AD (Grothe et al., 2018; Li et al., 2021). Consequently, we hypothesized that the underlying biological foundation of connectome susceptibility in AD could potentially find elucidation through neuroimaging genomics.

**FIGURE 1:**
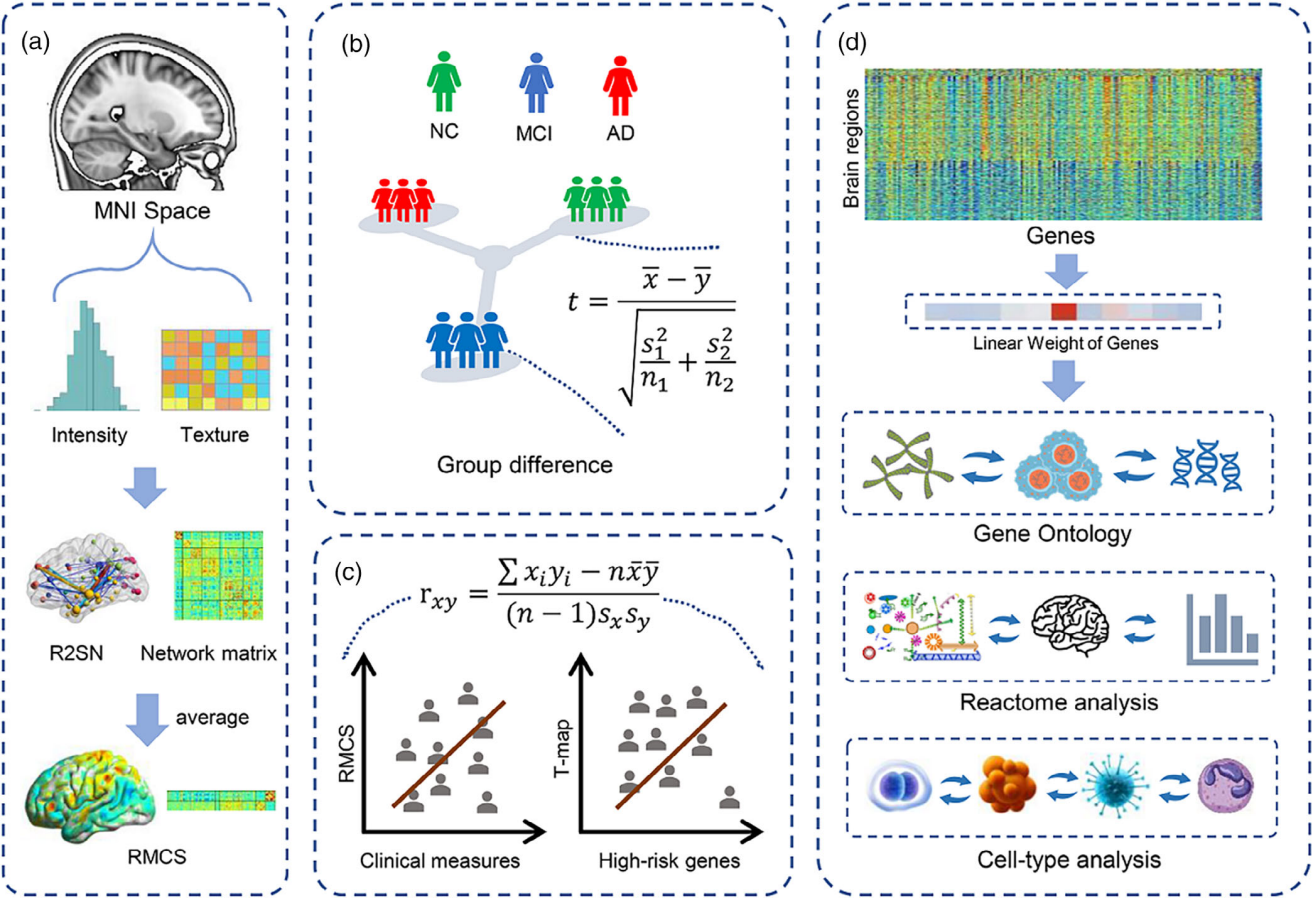
Schematic of the data analysis pipeline. (a) The construction of R2SN. (b) Group difference analysis of the RMCS among the NC, MCI, and AD groups. (c) Correlation analysis between RMCS and clinical measures/AD high‐risk genes. (d) Gene ontology, Reactome, and cell‐type analysis.

Therefore, in the present study, we speculated that the biological basis of the morphological connectivity differences in AD might be elucidated by combining neuroimaging and genetic (and microscale brain organization). We first introduced a regional morphological connectivity index entitled regional mean connectivity strength (RMCS) derived from the regional radiomics similarity network (R2SN; Zhao et al., 2021) to capture the brain structural connectome changes in AD. Then, we explored the relationship between gene expression and RMCS changes in the brain. Furthermore, to explore the molecular mechanisms of the morphological connectivity differences in AD, we also evaluated the association between the RMCS changes and cell‐type signals (Figure 1).

## 2 MATERIALS AND METHODS

### 2.1 Subjects

A total of 1654 participants (605 normal controls [NCs], 766 mild cognitive impairment [MCI], and 283 AD) from the Alzheimer's Disease Neuroimaging Initiative dataset (ADNI; https://adni.loni.usc.edu/ ) were included in the present study. Detailed information can be found in Table 1. Additional information about the ADNI dataset can be found at http://adni.loni.usc.edu/wpcontent/uploads/how_to_apply/ADNI_Acknowledgement_List.pdf.

**TABLE 1:** Detailed information on the subjects.

	Group	Age (years)	Sex (M/F)	Clinical measure
Subjects an MMSE (*N* = 1654)	NC (605)	73.47 ± 6.16	279/326	29.08 ± 1.10
	MCI (766)	72.96 ± 7.69	450/316	27.57 ± 1.81
	AD (283)	74.91 ± 7.70	152/131	23.18 ± 2.14
	*p*	.002	<.001	<.001
Subjects with an ADAS‐cog 11 (*N* = 1650)	NC (603)	73.49 ± 6.15	278/325	7.00 ± 3.04
	MCI (765)	72.98 ± 7.68	449/316	10.41 ± 4.42
	AD (282)	74.88 ± 7.70	151/131	19.65 ± 6.66
	*p*	.002	<.001	<.001
Subjects with an ADAS‐cog 13 (*N* = 1642)	NC (602)	73.51 ± 6.15	278/324	10.38 ± 4.37
	MCI (762)	72.97 ± 7.69	448/314	16.64 ± 6.66
	AD (278)	74.93 ± 7.66	148/130	30.03 ± 7.91
	p	.002	<.001	<.001
Subjects with an AVLT‐Im (*N* = 1649)	NC (603)	73.46 ± 6.17	278/325	45.34 ± 9.95
	MCI (766)	72.96 ± 7.69	450/316	34.52 ± 10.76
	AD (280)	74.83 ± 7.69	149/131	23.09 ± 7.54
	*p*	.003	<.001	<.001
Memory (*N* = 1652)	NC (605)	73.47 ± 6.16	279/326	1.01 ± 0.55
	MCI (764)	72.97 ± 7.69	448/316	0.18 ± 0.68
	AD (283)	74.90 ± 7.70	152/131	−0.86 ± 0.55
	*p*	.002	<.001	<.001
Executive (*N* = 1649)	NC (603)	73.49 ± 6.14	279/324	0.90 ± 0.82
	MCI (764)	72.97 ± 7.69	448/316	0.22 ± 0.91
	AD (282)	74.87 ± 7.69	151/131	−0.92 ± 0.94
	*p*	.002	<.001	<.001

*Note*: Data are presented as mean ± standard deviation for continuous variables. Independent ANOVA test for continuous variables and chi‐square test for categorical variables. Significance *p* < .05 with Bonferroni correction (*n* = 27).

### 2.2 Data acquisition and processing

For each participant, the structural MRI with T1‐weighted was aligned to Montreal Neurological Institute space by Advanced Normalization Tools with “SyN” parameters (Avants et al., 2009) after N4 bias correction and imaging denoise. In this study, for each region, 47 radiomics features were evaluated. Initially, a conventional min‐max approach was employed to normalize the radiomics features across different brain regions within an individual. Detailed descriptions of radiomics features can be found in Table S1 or elsewhere in our previous studies (Feng et al., 2018; Zhao et al., 2022). Subsequently, the traits that exhibited a strong correlation with other characteristics were identified as redundant features (*R* > 0.9; Table S2). The Brainnetome atlas defined the nodes of the R2SN, including 246 regions (Table S3; Fan et al., 2016), and the edges were assessed by the Pearson correlation between the radiomics features of each pair of brain regions after the min–max feature normalization (Zhao et al., 2021). This study quantifies the regional structural connectome as RMCS based on the R2SN.

### 2.3 Statistical analysis

To quantify the atypicality of the structural connectome in individuals with MCI and AD, we computed the difference in the RMCS among the AD, MCI, and NC groups after removing the influence of age and sex by using linear regression. First, we performed an analysis of variance (ANOVA) analysis on the RMCS values across 246 brain regions. Then, a two‐sided *t*‐test was employed to study the difference in RMCS in AD versus NC, MCI versus NC, and AD versus MCI, respectively. To further explore the robustness of the difference analysis, we iteratively selected subgroups within the AD, MCI, and NC groups, ensuring that age did not show significant differences among the groups (1000 times). After that, we computed the Pearson correlation between the statistical significance resulting from the random selections and the outcomes of the initial ANOVA analysis.

Additionally, to explore the clinical basis of those structural connectome changes, we also assessed the relationship between the RMCS and clinical measures, including Alzheimer's Disease Assessment Scale‐cognitive subscale (ADAS‐cog11, ADAS‐cog13), Auditory Verbal Learning Test‐Immediate Recall (AVLT1), Minimum Mental State Examination (MMSE), Memory and Executive ability in the MCI and AD groups, respectively.

### 2.4 Regional changes in RMCS and gene expression

To access the relation between gene expression and altered structural connectome in AD, we utilized Partial Least Squares (PLS) analysis to investigate the association between the T‐map of RMCS in AD versus NC and gene expression, with the former serving as the independent variable and the latter as the dependent variable. Briefly, the gene expression data are downloaded from the Allen atlas (six young participants, Table S9), which was drawn from six postmortem brains of donors without any neuropathological or neuropsychiatric diseases (http://human.brain-map.org/; Shen et al., 2012) and was subsequently projected onto the Brainnetome Atlas using the “abagen” toolkit (https://github.com/rmarkello/abagen). Ultimately, it yielded a gene expression dataset comprised of 15,633 genes obtained from 236 brain regions of Brainnetome Atlas (the list of 10 brain regions for which no gene information was listed in Table S4). In the present study, to ensure the specificity of our genetic results, we introduced a variogram‐based spatial auto‐correlation null model via the BrainSMASH python toolbox (https://brainsmash.readthedocs.io/en/latest/index.html; Burt et al., 2020). Importantly, to investigate the robustness of the result of gene enrichment, we conducted replication experiments between gene expression and the T‐map of RMCS in MCI versus NC and significant brain regions in AD versus NC following the same procedure.

### 2.5 Gene enrichment analysis

The PLS analysis effectively sorted the vast cohort of 15,633 genes based on their corresponding weight values. We selected the top 500 genes based on the sorting of gene weights by absolute value (Ackermann & Strimmer, 2009; Alexeyenko et al., 2012; Luo et al., 2009; Subramanian et al., 2005). After that, gene‐set enrichment analysis was performed based on the top 500 genes via the Metascape platform (https://metascape.org/gp/index.html#/main/step1; Zhou et al., 2019). Besides, to further explore the robustness of the gene enrichment, we also calculated gene‐set enrichment analysis for the top 1000 genes and assessed the replicability of these results.

Based on the list of characterized genes provided on the AHBA website (https://help.brain-map.org/display/humanbrain/Documentation), we found a set of 28 high‐risk genes related to AD, including A2M, ACE, ACHE, APBA1, APBB2, APLP1, APLP2, APOC1, APP, BACE2, BCHE, BLMH, CASP3, CHRNA3, CTSB, DBN1, ESR1, GSK3B, IL1B, KCNIP3, KLK6, LRP1, LRRC15, MAPT, PLAU, PSEN1, PSEN2, and SORL1 (Table S5). We also computed the Pearson correlation between those genes and the T‐map of the RMCS in AD versus NC.

### 2.6 Assigning cell type‐specific gene expression patterns

To further investigate the biological basis of RMCS changes in AD, we categorized cell types into seven distinct groups, as previously outlined in a study: (I) microglia; (II) endothelial cells; (III) oligodendrocyte precursors; (IV) oligodendrocytes; (V) astrocytes; (VI) inhibitory neurons; and (VII) excitatory neurons (Seidlitz et al., 2020). Subsequently, we overlapped the gene sets of each cell type with AD‐related genes to derive corresponding gene lists for each cell type. We determined the mean expression of each cell‐type gene set in the Brainnetome Atlas and normalized it concerning the entire brain, generating a 236‐region by seven‐cell matrix. We then computed the relationship between the cell type‐specific expression and the T‐map of the RMCS in AD versus NC. We also performed a spatial auto‐correlation method based on a variogram to observe the significance of the cell types.

## 3 RESULTS

### 3.1 Demographic and clinical characteristics

Among the NC, MCI, and AD groups, there was a significant difference in the age and sex ratio of the subjects. In addition, the clinical measures, including MMSE score, ADAS‐cog11 score, ADAS‐cog13 score, AVLT1 score, Executive and Memory ability were significantly different among the NC, MCI, and AD groups (*p* < .05, Bonferroni correction with *N* = 27).

### 3.2 RMCS showed significant differences in disease states

**FIGURE 2:**
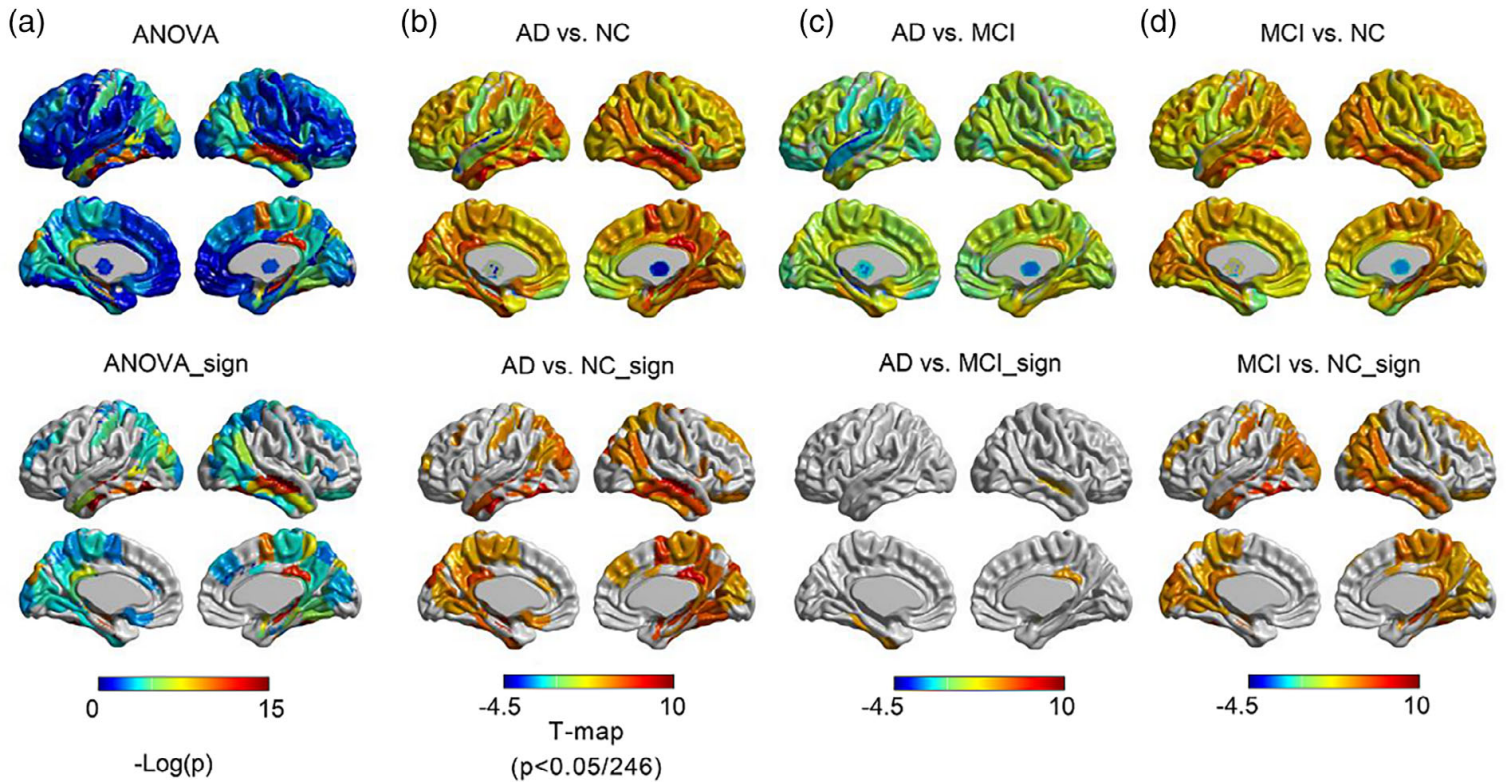
ANOVA analysis and group difference analysis. (a) Difference results of RMCS with ANOVA analysis. Group differences of RMCS in (b) AD versus NC, (c) AD versus MCI, and (d) MCI versus NC. The bottom row (with a suffix sign) represents the significant brain regions of *p* < .05/246. *T* value >0 represents a higher value of RMCS in AD.

ANOVA analysis showed that the RMCS was significantly differed in temporal, occipital, parietal, cingulate, and hippocampal among the NC, MCI, and AD groups (Figure 2a). The results of 1000 bootstrap analysis showed a high correlation with the original ANOVA analysis (*R* > 0.5 in 97.6% of 1000 times random selection), which further provides robust substantiation for the dependability and consistency of our experimental outcomes within this study (Figure S3). Subsequent two‐sided *t*‐test analysis identified the altered RMCS in the AD group's temporal lobe, posterior cingulate, and hippocampus (Figure 2b). Significant changes in RMCS in the temporal, occipital, and frontal lobes were observed in the MCI group but weaker than those in the AD group (Figure 2d; *p* < .05, Bonferroni correction with *N* = 246).

### 3.3 RMCS is associated with clinical measures

**FIGURE 3:**
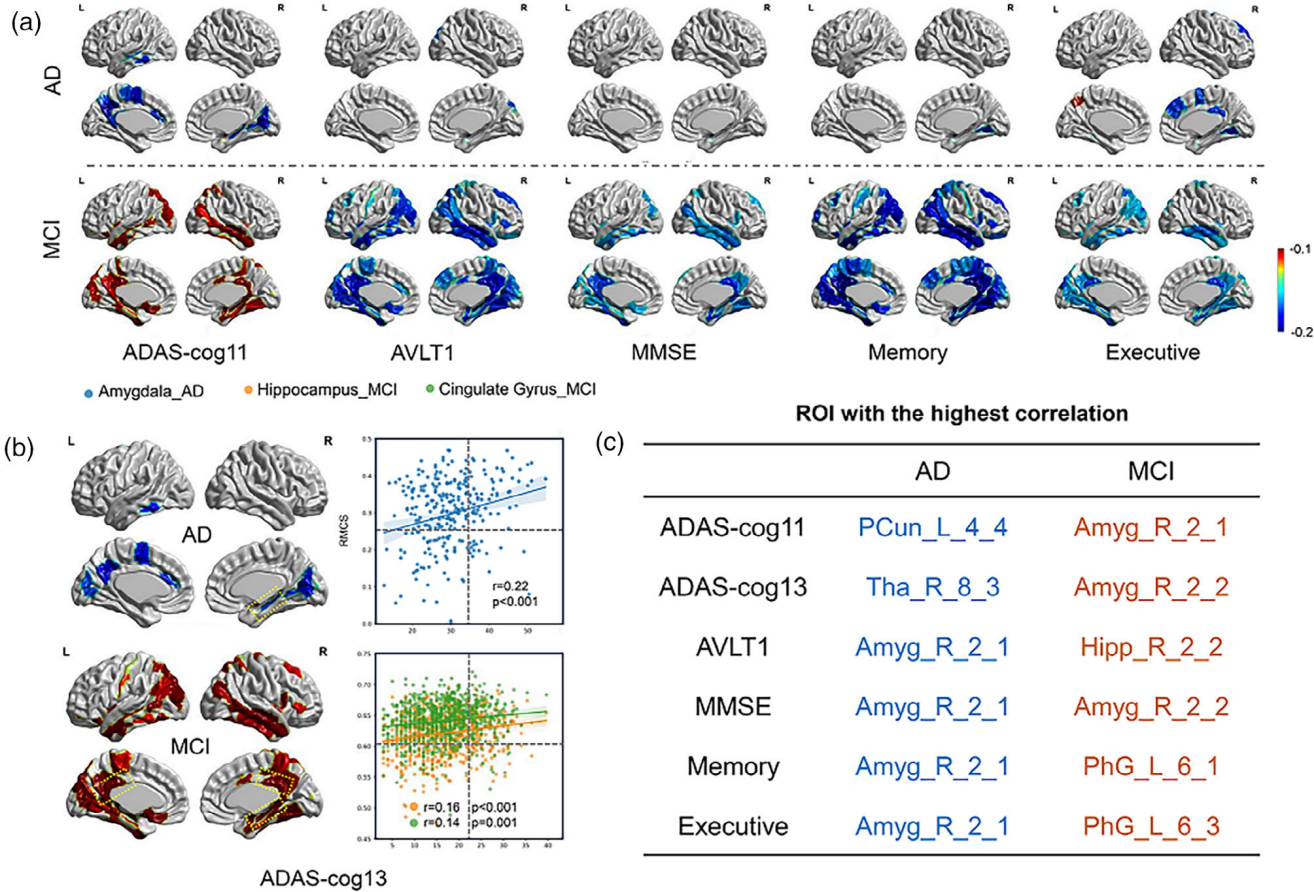
The results of the correlation between RMCS of the significant brain regions in (a) AD and MCI groups and clinical measures, including ADAS‐cog11, AVLT1, MMSE, Memory and Executive ability, and (b) ADAS‐cog13. (c) The representative brain regions with the highest correlation between RMCS and clinical measures.

As anticipated, the RMCS was significantly correlated with various clinical measures, especially in memory abilities. The amygdala, hippocampus, and cingulate gyrus still showed significant correlations with the clinical measures in MCI and AD groups (*p* < .05, no Bonferroni correction; Figure 3).

### 3.4 Enrichment pathways associated with changes in RMCS

**FIGURE 4:**
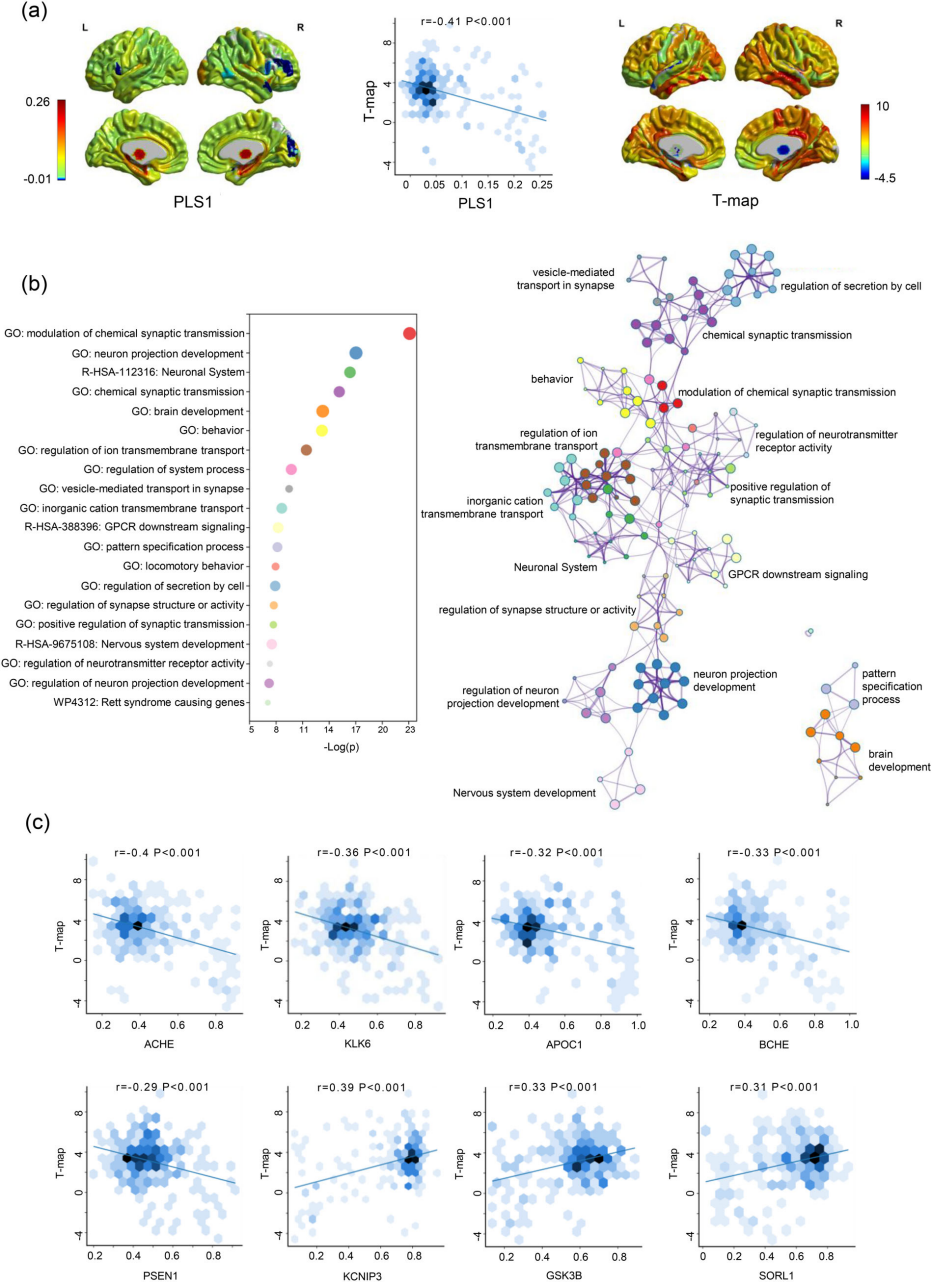
Functional enrichment of gene transcripts. (a) The correlation between the PLS1 score and the T‐map of the RMCS in AD versus NC. (b) The results of GO term and Reactome pathway. The size of the node is proportional to the number of input genes contained in the term, and its color represents cluster identity. (c) The correlation between the AD‐related genes and the T‐map of the RMCS in AD versus NC.

Given that the first PLS component (PLS1) comprehensively explains the most significant changes between the two sets of variables, we only employed PLS1 rather than other components in the following analysis (Table S7). Within this study, we found that the PLS1 accounted for 16.84% of the variability in gene expression and correlated with the T‐map of RMCS in AD versus NC (*r* = −.41, *p* < .001) (Figure 4a). The correlation value was more significant than spatially auto‐correlated null distributions of 10,000 repetitions (*p* < .001; Figure S2a). In addition, our gene enrichment analysis revealed that several GO biological processes, including modulation of chemical synaptic transmission (GO:0050804, *p* = 8.13e‐24), neuron projection development (GO:0031175, *p* = 9.33e‐18), chemical synaptic transmission (GO:0007268, *p* = 7.24e‐16), and brain development (GO:0007420, *p* = 5.37e‐14), were significantly enriched. The remaining gene enrichment results are provided in Table S6. Furthermore, Reactome pathway analysis indicated that the neuronal system (R‐HSA‐112316, p = 4.47e‐17), GPCR downstream signaling (R‐HSA‐388396, *p* = 5.89e‐9), and nervous system development pathways (R‐HSA‐9675108, *p* = 3.09e‐8) showed a significant correlation with brain structural connectomes in AD (Figure 4b). We found similar results from the top 1000 gene enrichment analysis (Figure S6).

Furthermore, the PLS1 was also significantly correlated with the T‐map of RMCS in MCI versus NC (*r* = −.49, *p* < .001) and with the T‐map of significant brain regions in AD versus NC (*r* = .51, *p* < .001). The correlation values were also more significant than spatially auto‐correlated null distributions of 10,000 repetitions (*p* < .001; Figures S4a and S7a). In addition, the weights of enriched genes in AD were highly consistent with that in MCI (*r* = .99, *p* < .001) and significant brain regions in AD (*r* = .99, *p* < .001; Figures S5 and S8). Specifically, we found significant enrichment in GO biological processes including modulation of chemical synaptic transmission (GO:0050804: MCI vs. NC, *p* = 2.45e‐15; significant brain regions in AD vs. NC, *p* = 5.75e‐16) and neuron projection development (GO:0031175: MCI vs. NC, *p* = 1.15e‐13; significant brain regions in AD vs. NC, *p* = 3.39e‐14) were repeatable in the PLS analysis based on T‐map of AD versus NC, T‐map of MCI versus NC and the significant regions of AD versus NC. Additionally, Reactome pathways such as the neuronal system (R‐HSA‐112316: MCI vs. NC, *p* = 1.70e‐15; significant brain regions in AD versus NC, *p* = 4.17e‐13) were also found to be significantly enriched in the PLS analysis based on T‐map of AD versus NC, T‐map of MCI versus NC and the significant regions of AD versus NC (Figures S4b and S7b). We also found the overlapping subset of the related genes (128/500) between the MCI and AD groups (Table S8).

Among 28 AD‐related genes, the ACHE, KLK6, APOC1, BCHE, and PSEN1 had strong negative, while KCNIP3, GSK3B, and SORL1 showed strong positive correlations with the T‐map of RMCS in AD compared to NC (*p* < .001; Figure 4c). The remaining 20 gene analysis results are provided in Figure S1.

### 3.5 Transcriptional signatures for typical cell types

**FIGURE 5:**
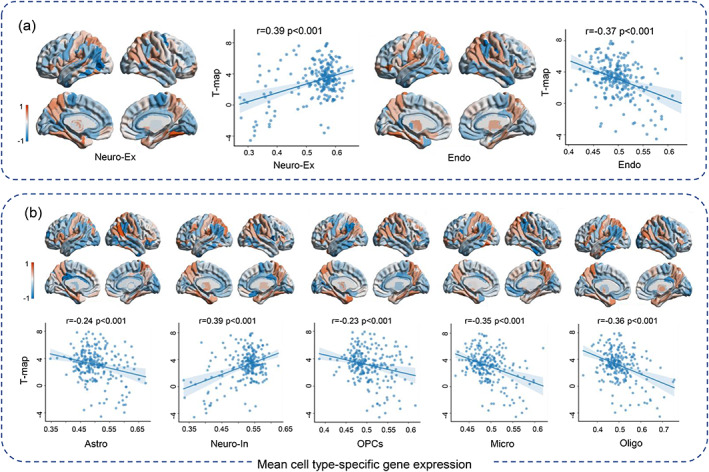
The results of associations between standardized cell type‐specific gene expression maps and T‐map of the RMCS in AD versus NC, including (a) Neuro‐Ex and Endo (b) Astro, Neuro‐In, OPCs, Micro and Oligo.

The correlation analysis demonstrates that the excitatory neurons (*r* = .39, *p* < .001) and inhibitory neurons (*r* = .39, *p* < .001) had a significant positive correlation with the T‐map of RMCS in AD and NC groups. Meanwhile, endothelial (*r* = −.37, *p* < .001) and oligodendrocytes (*r* = −.36, *p* < .001) were found to exhibit a strong negative correlation (Figure 5). The relationship between these cell types and the T‐map of RMCS in AD versus NC was significant compared to the spatially auto‐correlated distributions after 10,000 rotations (*p* < .001), except astrocytes (*p* = .0053) and oligodendrocyte precursors (*p* = .0056; Figure S2b).

## 4 DISCUSSION

This study systematically demonstrates that RMCS derived from R2SN could serve as a new index for representing the brain structural changes in AD. Moreover, radiogenomics analysis also bridges gaps in the biological basis of RMCS captured by R2SN. We investigated its relationship with cognitive ability and gene expression. Remarkably, we observed significant correlations between the RMCS and these biological measures. This result suggests that the RMCS may reflect changes in cognitive abilities, AD‐associated gene expression, and cell type. Therefore, these findings provide a theoretical foundation for applying R2SN to understand the structural connectome changes in brain disorders.

The consensus within the scientific community is that AD is characterized by disconnection throughout the brain rather than isolated regions of damage (Alexander‐Bloch et al., 2013; Delbeuck et al., 2003). R2SN provides a comprehensive approach to characterize regional‐based brain structure alterations and depict global brain changes (Zhao et al., 2021). However, despite the advancements made by R2SN, there is still a gap in understanding the biological basis for the alterations observed in AD. Genetic transcriptomics serves as the foundational framework for understanding biological variability. Integrating transcriptomic data with extensive morphometric networks enhances our understanding of how these genetic components intricately shape modifications in both brain structure and function (Li et al., 2021), with a pronounced focus on the context of AD. This integration, in turn, offers a novel insight into AD mechanisms from a connectome perspective. Our recent studies have demonstrated that R2SN can accurately capture the structural connectome changes in AD (Zhao et al., 2023; Zhao et al., 2021; Zhao et al., 2022). Additionally, our findings show a correlation between RMCS and cognitive ability and microstructural genetics, further emphasizing the significance of investigating brain connectome changes in other disorders using R2SN.

This study highlights the importance of the ACHE gene among the genes associated with AD, as it appears to have the most significant impact on structural connectome alteration in the brain. ACHE and KCNIP3, the two most significantly correlated genes, have demonstrated a strong association with AD (Anekonda et al., 2011; Galimberti & Scarpini, 2016). The primary function of the ACHE is to degrade acetylcholine and terminate neurotransmission (Murray et al., 2013). Additionally, the metabolism of acetylcholine is regulated by BCHE and can lead to decreased enzyme activity and cortical acetylcholine levels, increasing AD risk (Darvesh, 2016; Jasiecki & Wasag, 2019). It is worth noting that prolonged overexpression of GSK3b can deplete neurogenic niches, leading to the emergence of common AD symptoms such as Tau phosphorylation, amyloid‐β production, and synaptic dysfunction (Hernandez & Avila, 2008; Lauretti et al., 2020; Proctor & Gray, 2010). Those results suggested that the expression of ACHE might be reflected in observable changes in the structural connectome, demonstrating a clear link between gene expression and macroscopic brain changes. Therefore, comprehending the interplay between gene function and their expression levels in the brain will provide valuable insights for AD research.

Our investigation into the genetic basis of structural connectome abnormalities in AD has provided new insight into the pathogenesis of this disorder. AD is considered a synaptic dysfunction disorder in which synaptic failure always occurs early (Chen et al., 2019). Synapse is the foundation of cognitive activity, and any neural reflex activity of the central nervous system affects synaptic transmission. In particular, the transmission of neurotransmitters through the synapse is crucial for neural reflex activity and cognitive function (Trinchese et al., 2008). We conducted gene enrichment analysis and found that the altered brain structure in AD is significantly enriched in biological pathways related to “modulation of chemical synaptic transmission”, “neuron projection development”, and “chemical synaptic transmission”. Brain development is also an essential related pathway within these biological processes that cannot be overlooked. It is an incredibly complex process, and normal brain development relies heavily on regulating neurons and their synapses (Farizatto & Baldwin, 2023). Dysregulation of multiple metabolic pathways involved in brain development or aging may play a significant role in developing AD‐related structural alterations and subsequent cognitive impairments (Loeffler‐Wirth et al., 2022). Further research and exploration of the underlying mechanisms linking brain development/aging and AD may provide valuable insights into the early detection and prevention of this devastating neurodegenerative disease. These findings provide important clues to the pathogenesis of AD, which may be related to the abnormal metabolic pathways of multiple molecules, ultimately leading to nervous system disorders and memory loss (Mahajan et al., 2020). Identifying these critical pathways is of great significance for understanding and treating AD.

Cells can be likened to the building blocks of human life, forming the foundation of human growth and development. A deeper understanding of the functions of specific cells can lead to improved approaches toward the various conditions presented in AD (Forcaia et al., 2021; Valenza et al., 2021). This study demonstrated that the excitatory and inhibitory neurons significantly correlated with the brain connectome changes in AD. The disruption in neuronal activity is a crucial factor underlying structural brain pathology and cognitive dysfunction. Extensive research has established the pivotal role of microglia and astrocytes in the neuroinflammation associated with AD (Carter et al., 2019; Hemonnot et al., 2019; Yamazaki et al., 2019). At the same time, there were also some previous studies have demonstrated the critical role of non‐neuronal cells, such as microglia, in constructing neural circuits' excitability and plasticity (Harris et al., 2020; Kunkle et al., 2019). The malignant interaction between non‐neuronal cells and neurons can lead to synaptic abnormalities, neuron loss, and eventual neural system failure in AD (Maestu et al., 2021). Therefore, these comprehensive findings support that R2SN captured the structural changes in AD and has a solid biological basis.

## 5 LIMITATION

There are several limitations to the present study. First, to confirm the biological basis of the observed changes in the structural connectome, it would be advantageous to conduct molecular imaging studies using positron emission tomography, for example, with AV1451 or AV45. Second, we should investigate whether the identified genes and cell types are specific to AD. Additionally, to enhance the reliability of our findings, it would be valuable to validate the robustness of our results in an independent dataset. Finally, we should note age differences between imaging and gene expression data as potentially impacting the results.

## 6 CONCLUSION

In this study, we captured morphological connectivity changes in the AD based on RMCS derived from R2SN and uncovered the biological mechanisms behind these changes. The modulation of chemical synaptic transmission is the most relevant biological process and the neurons are the most relevant cells. In summary, our findings enhance our understanding of the intricate relationship between genetics, morphological changes, and the progression of disease. Importantly, these insights have the potential to provide valid reference value for the development of more accurate diagnostic tools and personalized therapeutic strategies for AD.

## AUTHOR CONTRIBUTIONS

Huiying Yu, Yanhui Ding, Dong Wang, Xiaopeng Kang, and Kun Zhao analyzed the data and performed the measurements; Huiying Yu, Kun Zhao, and Yong Liu were principally responsible for preparing the manuscript; Yongbin Wei, Martin Dyrba, Weizhi Xu, Kun Zhao, and Yong Liu revised the manuscript; Yong Liu supervised the project.

## CONFLICT OF INTEREST STATEMENT

The authors declare no conflict of interest

## Supporting information


**Data S1:** Supporting information

## Data Availability

The data that support the findings of this study are available in Alzheimer's Disease Neuroimaging Initiative dataset (ADNI) at https://adni.loni.usc.edu/.
