# Supplementary material for: Morphological connectivity differences in Alzheimer's disease correlate with gene transcription and cell‐type
Source: Hum Brain Mapp. 2023 Oct 17;44(18):6364–74. doi: 10.1002/hbm.26512 (PMC10681645; doi:10.1002/hbm.26512)
Supplement: Supplementary file 1 — Data S1: Supporting information [file HBM-44-6364-s001.docx]

Morphological connectivity differences in Alzheimer’s disease correlate with gene transcription and cell-type

**Table S1.** The specific information of a total of 47 features extracted from MRI, including intensity features and texture features, was list here.

|  | **Image feature** | **Equation** | **Definition** |
| --- | --- | --- | --- |
| Intensity features (14) | Energy | $\sum_{i}^{N} {X(i)}^{2}$ | Measure of the randomness of the intensity values in an image |
|  | Entropy | $\sum_{i=1}^{N_{l}} P\left( i \right)\log_{2}P\left( i \right)$ | Represents the irregularity of the intensity value distribution |
|  | Kurtosis | $\frac{\frac{1}{N}\sum_{i=1}^{N} \left( X\left( i \right)-\bar{X} \right)^{4}}{\left( \sqrt{\frac{1}{N}\sum_{i=1}^{N} \left( X\left( i \right)-\bar{X} \right)^{2}} \right)^{2}}$ | The peakedness of the histogram or indication of histogram flatness |
|  | Maximum | Maximum intensity value of X |  |
|  | Mean | $\frac{1}{N}\sum_{i}^{N} X(i)$ | Average intensity value of the pixels within the region of interest |
|  | Mean Absolute Deviation (mad) | Mean of the absolute deviations of all voxel intensities around the mean intensity value | A measure of how much the gray levels differ from the mean |
|  | Median | Median intensity value of X |  |
|  | Minimum | Minimum intensity value of X |  |
|  | Range | Range of intensity values of X |  |
|  | Root Mean Square (RMS) | $\sqrt{\frac{{\sum_{i}^{N} X(i)}^{2}}{N}}$ |  |
|  | Skewness | $\frac{\frac{1}{N}\sum_{i=1}^{N} {(X\left( i \right)-\bar{X})}^{3}}{\left( \sqrt{\frac{1}{N}\sum_{i=1}^{N} {(X\left( i \right)-\bar{X})}^{2}} \right)^{3}}$ | Symmetry of intensity values in an image |
|  | Standard Deviation | $\left( \frac{1}{N-1}\sum_{i=1}^{N} \left( X\left( i \right)-\bar{X} \right)^{2} \right)^{1/2}$ | A measure of how much variation or dispersion exists |
|  | Uniformity | $\sum_{i=1}^{N_{l}} {P(i)}^{2}$ | Measures the homogeneity of the intensity value distribution in an image |
|  | Variance (Var) | $\frac{1}{N-1}\sum_{i=1}^{N} \left( X\left( i \right)-\bar{X} \right)^{2}$ | The spread or variation around the mean (sum of squares) |

**X** denotes the three-dimensional image matrix. ***N*** is the number of voxels. **P** is the first-order histogram with ***N_l_*** discrete intensity levels.‾X is the mean of X. The number of histogram bins is 100.

|  | Image feature | Equation | Definition |  |
| --- | --- | --- | --- | --- |
| Textural features (33) | Autocorrelation | $\sum_{i=1}^{N_{g}} \sum_{j=1}^{N_{g}} ijP(i,j)$ |  |  |
|  | Cluster Prominence (CP) | $\sum_{i=1}^{N_{g}} \sum_{j=1}^{N_{g}} \left[ i+j-\mu_{x}\left( i \right)-\mu_{y}\left( j \right) \right]^{4}P(i,j)$ |  |  |
|  | Cluster Shade | $\sum_{i=1}^{N_{g}} \sum_{j=1}^{N_{g}} \left[ i+j-\mu_{x}\left( i \right)-\mu_{y}\left( j \right) \right]^{3}P(i,j)$ |  |  |
|  | Cluster Tendency | $\sum_{i=1}^{N_{g}} \sum_{j=1}^{N_{g}} \left[ i+j-\mu_{x}\left( i \right)-\mu_{y}\left( j \right) \right]^{2}P(i,j)$ |  |  |
|  | Contrast | $\sum_{i=1}^{N_{g}} \sum_{j=1}^{N_{g}} \left\vert i-j \right\vert^{2}P(i,j)$ | Measures the local variation in intensity values |  |
|  | Correlation | $\frac{\sum_{i=1}^{N_{g}} \sum_{j=1}^{N_{g}} ijP\left( i,j \right)-\mu_{i}(i)\mu_{j}(j)}{\sigma_{x}\left( i \right)\sigma_{y}(j)}$ | Measures the linear dependencies of intensity values in an image |  |
|  | Difference Entropy | $\sum_{i=0}^{N_{g}-1} P_{x-y}\left( i \right){log}_{2}\left[ P_{x-y}(i) \right]$ |  |  |
|  | Dissimilarity | $\sum_{i=1}^{N_{g}} \sum_{j=1}^{N_{g}} \left\vert i-j \right\vert P(i,j)$ |  |  |
|  | Energy | $\sum_{i=1}^{N_{g}} \sum_{j=1}^{N_{g}} \left[ P(i,j) \right]^{2}$ |  |  |
|  | Entropy | $-\sum_{i=1}^{N_{g}} \sum_{j=1}^{N_{g}} P\left( i,j \right){log}_{2}\left[ P\left( i,j \right) \right]$ |  |  |
|  | Homogeneity1 | $\sum_{i=1}^{N_{g}} \sum_{j=1}^{N_{g}} \frac{P\left( i,j \right)}{1+\left\vert i-j \right\vert}$ | Measures the homogeneity of the intensity values |  |
|  | Homogeneity2 | $\sum_{i=1}^{N_{g}} \sum_{j=1}^{N_{g}} \frac{P\left( i,j \right)}{1+\left\vert i-j \right\vert^{2}}$ | Measures the homogeneity of the intensity values of the pixel pair | |
|  | Informational Measure of Correlation 1 (IMC1) | $\frac{HXY-HXY1}{max\left\{ HX,HY \right\}}$ |  | |
|  | Informational Measure of Correlation 2 (IMC2) | $\sqrt{1-e^{-2\left( HXY2-HXY \right)}}$ |  | |
|  | Inverse Difference Moment Normalized (IDMN) | $\sum_{i=1}^{N_{g}} \sum_{j=1}^{N_{g}} \frac{P\left( i,j \right)}{1+\left( \frac{\left\vert i-j \right\vert^{2}}{N^{2}} \right)}$ |  | |
|  | Inverse Difference Normalized (IDN) | $\sum_{i=1}^{N_{g}} \sum_{j=1}^{N_{g}} \frac{P\left( i,j \right)}{1+\left( \frac{\left\vert i-j \right\vert}{N} \right)}$ |  | |
|  | Inverse Variance | $\sum_{i=1}^{N_{g}} \sum_{j=1}^{N_{g}} \frac{P\left( i,j \right)}{\left\vert i-j \right\vert^{2}}, i\neq j$ |  | |
|  | Maximum Probability | max$\left\{ P\left( i,j \right) \right\}$ |  | |
|  | Sum Average | $\sum_{i=2}^{{2N}_{g}} \left[ iP_{x+y}(i) \right]$ |  | |
|  | Sum Entropy | $-\sum_{i=2}^{{2N}_{g}} P_{x+y}(i){log}_{2}\left[ P_{x+y}(i) \right]$ |  | |
|  | Sum Variance | $\sum_{i=2}^{{2N}_{g}} \left( i-SE \right)^{2}P_{x+y}(i)$ |  | |
|  | Variance | $\sum_{i=1}^{N_{g}} \sum_{j=1}^{N_{g}} \left( i-\mu\right)^{2}P(i,j)$ |  | |
|  | Short Run Emphasis (SRE) | $\frac{\sum_{i=1}^{N_{g}} \sum_{j=1}^{N_{r}} \left[ \frac{p\left( i,j \vert\theta\right)}{j^{2}} \right]}{\sum_{i=1}^{N_{g}} \sum_{j=1}^{N_{r}} p\left( i,j \vert\theta\right)}$ |  | |
|  | Long Run Emphasis (LRE) | $\frac{\sum_{i=1}^{N_{g}} \sum_{j=1}^{N_{r}} j^{2}p\left( i,j \vert\theta\right)}{\sum_{i=1}^{N_{g}} \sum_{j=1}^{N_{r}} p\left( i,j \vert\theta\right)}$ |  | |
|  | Gray Level Nonuniformity (GLN) | $\frac{\sum_{i=1}^{N_{g}} \left[ \sum_{j=1}^{N_{r}} p\left( i,j \vert\theta\right) \right]^{2}}{\sum_{i=1}^{N_{g}} \sum_{j=1}^{N_{r}} p\left( i,j \vert\theta\right)}$ | Represents the similarity of intensity values in an image | |
|  |  |  |  | |

$P(i,j)$ is the co-occurrence matrix for an arbitrary $\delta$ and$\alpha$

$N_{g}$is the number of discrete intensity levels in the image

$p\left( i,j | \theta\right)$ is the $\left( i,j \right)$th entry in the given run-length matrix $p$ for a direction $\theta$

*N_g_* is the number of discrete intensity values in the image

*N_r_* is the number of different run lengths

*N_p_* is the number of voxels in the image

u is the mean of $P(i,j)$

$p_{x}(i)$=$\sum_{j=1}^{N_{g}} P\left( i,j \right)$ is the marginal row probabilities

$p_{y}(i)$=$\sum_{i=1}^{N_{g}} P\left( i,j \right)$ is the marginal column probabilities

$\mu_{x}$ is the mean of$p_{x}$

$\mu_{y}$ is the mean of$p_{y}$

$\sigma_{x}$is the standard deviation of$p_{x}$

$\sigma_{y}$is the standard deviation of$p_{y}$

$p_{x+y}\left( k \right)$=$\sum_{i=1}^{N_{g}} \sum_{j=1}^{N_{g}} P\left( i,j \right)$, i$+$j=k, k=2,3,…,2*$N_{g}$

$P_{x-y}\left( k \right)$=$\sum_{i=1}^{N_{g}} \sum_{j=1}^{N_{g}} P\left( i,j \right)$,$\left| i-j \right|$=k, k=0,1,…,$N_{g}-$1

HX=$-\sum_{i=1}^{N_{g}} p_{x}\left( i \right)\log_{2}\left[ p_{x}\left( i \right) \right]$ is the entropy of $p_{x}$

HY=$-\sum_{i=1}^{N_{g}} p_{y}\left( i \right)\log_{2}\left[ p_{y}\left( i \right) \right]$ is the entropy of $p_{y}$

H=$-\sum_{i=1}^{N_{g}} \sum_{j=1}^{N_{g}} P\left( i,j \right)\log_{2}\left[ P\left( i,j \right) \right]$ is the entropy of *P*$\left( i,j \right)$

HXY1=$-\sum_{i=1}^{N_{g}} \sum_{j=1}^{N_{g}} P\left( i,j \right)log\left( p_{x}\left( i \right)p_{y}\left( j \right) \right)$

HXY2=$-\sum_{i=1}^{N_{g}} \sum_{j=1}^{N_{g}} p_{x}\left( i \right)p_{y}\left( j \right)log\left( p_{x}\left( i \right)p_{y}\left( j \right) \right)$

After the features that were highly correlated with other features were defined as redundant features were removed (R>0.9), a final feature matrix of 25x246 for each subject was obtained for further analysis([Zhao et al., 2021](#_ENREF_2)).

**Table S2.** The reserved features after removing superfluous features

| **Intensity features** | energy |
| --- | --- |
|  | kurtosis |
|  | maximum |
|  | mad |
|  | minimum |
|  | skewness |
|  | entropy |
| **Textural features** | Autocorrelation |
|  | Cluster Prominence |
|  | Cluster Shade |
|  | Cluster Tendency |
|  | Contrast |
|  | Correlation |
|  | Energy |
|  | Entropy |
|  | Homogeneity1 |
|  | IMC1 |
|  | Maximum Probability |
|  | Sum Entropy |
|  | Short Run Emphasis |
|  | Long Run Emphasis |
|  | Gray Level Nonuniformity |
|  | Low Gray Level Run Emphasis |
|  | High Gray Level Run Emphasis |
|  | Long Run High Gray Level Emphasis |

**Table S3.** The detailed brain regions’ names of the Brainnetome atlas (<https://atlas.brainnetome.org/>) ([Fan et al., 2016](#_ENREF_1)).

| **Lobe** | **Gyrus** | **Left and Right Hemisphere** | **Label ID.L** | **Label ID.R** | **Anatomical and modified Cyto-architectonic descriptions** |
| --- | --- | --- | --- | --- | --- |
| **Frontal Lobe** | SFG, Superior Frontal Gyrus | SFG_L(R)_7_1 | 1 | 2 | *A8m, medial area 8* |
|  |  | SFG_L(R)_7_2 | 3 | 4 | *A8dl, dorsolateral area 8* |
|  |  | SFG_L(R)_7_3 | 5 | 6 | *A9l, lateral area 9* |
|  |  | SFG_L(R)_7_4 | 7 | 8 | *A6dl, dorsolateral area 6* |
|  |  | SFG_L(R)_7_5 | 9 | 10 | *A6m, medial area 6* |
|  |  | SFG_L(R)_7_6 | 11 | 12 | *A9m,medial area 9* |
|  |  | SFG_L(R)_7_7 | 13 | 14 | *A10m, medial area 10* |
|  | MFG, Middle Frontal Gyrus | MFG_L(R)_7_1 | 15 | 16 | *A9/46d, dorsal area 9/46* |
|  |  | MFG_L(R)_7_2 | 17 | 18 | *IFJ, inferior frontal junction* |
|  |  | MFG_L(R)_7_3 | 19 | 20 | *A46, area 46* |
|  |  | MFG_L(R)_7_4 | 21 | 22 | *A9/46v, ventral area 9/46* |
|  |  | MFG_L(R)_7_5 | 23 | 24 | *A8vl, ventrolateral area 8* |
|  |  | MFG_L(R)_7_6 | 25 | 26 | *A6vl, ventrolateral area 6* |
|  |  | MFG_L(R)_7_7 | 27 | 28 | *A10l, lateral area10* |
|  | IFG, Inferior Frontal Gyrus | IFG_L(R)_6_1 | 29 | 30 | *A44d,dorsal area 44* |
|  |  | IFG_L(R)_6_2 | 31 | 32 | *IFS, inferior frontal sulcus* |
|  |  | IFG_L(R)_6_3 | 33 | 34 | *A45c, caudal area 45* |
|  |  | IFG_L(R)_6_4 | 35 | 36 | *A45r, rostral area 45* |
|  |  | IFG_L(R)_6_5 | 37 | 38 | *A44op, opercular area 44* |
|  |  | IFG_L(R)_6_6 | 39 | 40 | *A44v, ventral area 44* |
|  | OrG, Orbital Gyrus | OrG_L(R)_6_1 | 41 | 42 | *A14m, medial area 14* |
|  |  | OrG_L(R)_6_2 | 43 | 44 | *A12/47o, orbital area 12/47* |
|  |  | OrG_L(R)_6_3 | 45 | 46 | *A11l, lateral area 11* |
|  |  | OrG_L(R)_6_4 | 47 | 48 | *A11m, medial area 11* |
|  |  | OrG_L(R)_6_5 | 49 | 50 | *A13, area 13* |
|  |  | OrG_L(R)_6_6 | 51 | 52 | *A12/47l, lateral area 12/47* |
|  | PrG, Precentral Gyrus | PrG_L(R)_6_1 | 53 | 54 | *A4hf, area 4(head and face region)* |
|  |  | PrG_L(R)_6_2 | 55 | 56 | *A6cdl, caudal dorsolateral area 6* |
|  |  | PrG_L(R)_6_3 | 57 | 58 | *A4ul, area 4(upper limb region)* |
|  |  | PrG_L(R)_6_4 | 59 | 60 | *A4t, area 4(trunk region)* |
|  |  | PrG_L(R)_6_5 | 61 | 62 | *A4tl, area 4(tongue and larynx region)* |
|  |  | PrG_L(R)_6_6 | 63 | 64 | *A6cvl, caudal ventrolateral area 6* |
|  | PCL, Paracentral Lobule | PCL_L(R)_2_1 | 65 | 66 | *A1/2/3ll, area1/2/3 (lower limb region)* |
|  |  | PCL_L(R)_2_2 | 67 | 68 | *A4ll, area 4, (lower limb region)* |
| **Temporal Lobe** | STG, Superior Temporal Gyrus | STG_L(R)_6_1 | 69 | 70 | *A38m, medial area 38* |
|  |  | STG_L(R)_6_2 | 71 | 72 | *A41/42, area 41/42* |
|  |  | STG_L(R)_6_3 | 73 | 74 | *TE1.0 and TE1.2* |
|  |  | STG_L(R)_6_4 | 75 | 76 | *A22c, caudal area 22* |
|  |  | STG_L(R)_6_5 | 77 | 78 | *A38l, lateral area 38* |
|  |  | STG_L(R)_6_6 | 79 | 80 | *A22r, rostral area 22* |
|  | MTG, Middle Temporal Gyrus | MTG_L(R)_4_1 | 81 | 82 | *A21c, caudal area 21* |
|  |  | MTG_L(R)_4_2 | 83 | 84 | *A21r, rostral area 21* |
|  |  | MTG_L(R)_4_3 | 85 | 86 | *A37dl, dorsolateral area37* |
|  |  | MTG_L(R)_4_4 | 87 | 88 | *aSTS, anterior superior temporal sulcus* |
|  | ITG, Inferior Temporal Gyrus | ITG_L(R)_7_1 | 89 | 90 | *A20iv, intermediate ventral area 20* |
|  |  | ITG_L(R)_7_2 | 91 | 92 | *A37elv, extreme lateroventral area37* |
|  |  | ITG_L(R)_7_3 | 93 | 94 | *A20r, rostral area 20* |
|  |  | ITG_L(R)_7_4 | 95 | 96 | *A20il, intermediate lateral area 20* |
|  |  | ITG_L(R)_7_5 | 97 | 98 | *A37vl, ventrolateral area 37* |
|  |  | ITG_L(R)_7_6 | 99 | 100 | *A20cl, caudolateral of area 20* |
|  |  | ITG_L(R)_7_7 | 101 | 102 | *A20cv, caudoventral of area 20* |
|  | FuG, Fusiform Gyrus | FuG_L(R)_3_1 | 103 | 104 | *A20rv, rostroventral area 20* |
|  |  | FuG_L(R)_3_2 | 105 | 106 | *A37mv, medioventral area37* |
|  |  | FuG_L(R)_3_3 | 107 | 108 | *A37lv, lateroventral area37* |
|  | PhG, Parahippocampal Gyrus | PhG_L(R)_6_1 | 109 | 110 | *A35/36r, rostral area 35/36* |
|  |  | PhG_L(R)_6_2 | 111 | 112 | *A35/36c, caudal area 35/36* |
|  |  | PhG_L(R)_6_3 | 113 | 114 | *TL, area TL (lateral PPHC, posterior parahippocampal gyrus)* |
|  |  | PhG_L(R)_6_4 | 115 | 116 | *A28/34, area 28/34 (EC, entorhinal cortex)* |
|  |  | PhG_L(R)_6_5 | 117 | 118 | *TI, area TI(temporal agranular insular cortex)* |
|  |  | PhG_L(R)_6_6 | 119 | 120 | *TH, area TH (medial PPHC)* |
|  | pSTS, posterior Superior Temporal Sulcus | pSTS_L(R)_2_1 | 121 | 122 | *rpSTS, rostroposterior superior temporal sulcus* |
|  |  | pSTS_L(R)_2_2 | 123 | 124 | *cpSTS, caudoposterior superior temporal sulcus* |
| **Parietal Lobe** | SPL, Superior Parietal Lobule | SPL_L(R)_5_1 | 125 | 126 | *A7r, rostral area 7* |
|  |  | SPL_L(R)_5_2 | 127 | 128 | *A7c, caudal area 7* |
|  |  | SPL_L(R)_5_3 | 129 | 130 | *A5l, lateral area 5* |
|  |  | SPL_L(R)_5_4 | 131 | 132 | *A7pc, postcentral area 7* |
|  |  | SPL_L(R)_5_5 | 133 | 134 | *A7ip, intraparietal area 7(hIP3)* |
|  | IPL, Inferior Parietal Lobule | IPL_L(R)_6_1 | 135 | 136 | *A39c, caudal area 39(PGp)* |
|  |  | IPL_L(R)_6_2 | 137 | 138 | *A39rd, rostrodorsal area 39(Hip3)* |
|  |  | IPL_L(R)_6_3 | 139 | 140 | *A40rd, rostrodorsal area 40(PFt)* |
|  |  | IPL_L(R)_6_4 | 141 | 142 | *A40c, caudal area 40(PFm)* |
|  |  | IPL_L(R)_6_5 | 143 | 144 | *A39rv, rostroventral area 39(PGa)* |
|  |  | IPL_L(R)_6_6 | 145 | 146 | *A40rv, rostroventral area 40(PFop)* |
|  | Pcun, Precuneus | PCun_L(R)_4_1 | 147 | 148 | *A7m, medial area 7(PEp)* |
|  |  | PCun_L(R)_4_2 | 149 | 150 | *A5m, medial area 5(PEm)* |
|  |  | PCun_L(R)_4_3 | 151 | 152 | *dmPOS, dorsomedial parietooccipital sulcus(PEr)* |
|  |  | PCun_L(R)_4_4 | 153 | 154 | *A31, area 31 (Lc1)* |
|  | PoG, Postcentral Gyrus | PoG_L(R)_4_1 | 155 | 156 | *A1/2/3ulhf, area 1/2/3(upper limb, head and face region)* |
|  |  | PoG_L(R)_4_2 | 157 | 158 | *A1/2/3tonIa, area 1/2/3(tongue and larynx region)* |
|  |  | PoG_L(R)_4_3 | 159 | 160 | *A2, area 2* |
|  |  | PoG_L(R)_4_4 | 161 | 162 | *A1/2/3tru, area1/2/3(trunk region)* |
| **Insular Lobe** | INS, Insular Gyrus | INS_L(R)_6_1 | 163 | 164 | *G, hypergranular insula* |
|  |  | INS_L(R)_6_2 | 165 | 166 | *vIa, ventral agranular insula* |
|  |  | INS_L(R)_6_3 | 167 | 168 | *dIa, dorsal agranular insula* |
|  |  | INS_L(R)_6_4 | 169 | 170 | *vId/vIg, ventral dysgranular and granular insula* |
|  |  | INS_L(R)_6_5 | 171 | 172 | *dIg, dorsal granular insula* |
|  |  | INS_L(R)_6_6 | 173 | 174 | *dId, dorsal dysgranular insula* |
| **Limbic Lobe** | CG, Cingulate Gyrus | CG_L(R)_7_1 | 175 | 176 | *A23d, dorsal area 23* |
|  |  | CG_L(R)_7_2 | 177 | 178 | *A24rv, rostroventral area 24* |
|  |  | CG_L(R)_7_3 | 179 | 180 | *A32p, pregenual area 32* |
|  |  | CG_L(R)_7_4 | 181 | 182 | *A23v, ventral area 23* |
|  |  | CG_L(R)_7_5 | 183 | 184 | *A24cd, caudodorsal area 24* |
|  |  | CG_L(R)_7_6 | 185 | 186 | *A23c, caudal area 23* |
|  |  | CG_L(R)_7_7 | 187 | 188 | *A32sg, subgenual area 32* |
| **Occipital Lobe** | MVOcC*,* MedioVentral Occipital Cortex | MVOcC _L(R)_5_1 | 189 | 190 | *cLinG, caudal lingual gyrus* |
|  |  | MVOcC _L(R)_5_2 | 191 | 192 | *rCunG, rostral cuneus gyrus* |
|  |  | MVOcC _L(R)_5_3 | 193 | 194 | *cCunG, caudal cuneus gyrus* |
|  |  | MVOcC _L(R)_5_4 | 195 | 196 | *rLinG, rostral lingual gyrus* |
|  |  | MVOcC _L(R)_5_5 | 197 | 198 | *vmPOS,ventromedial parietooccipital sulcus* |
|  | LOcC, lateral Occipital Cortex | LOcC_L(R)_4_1 | 199 | 200 | *mOccG, middle occipital gyrus* |
|  |  | LOcC _L(R)_4_2 | 201 | 202 | *V5/MT+, area V5/MT+* |
|  |  | LOcC _L(R)_4_3 | 203 | 204 | *OPC, occipital polar cortex* |
|  |  | LOcC_L(R)_4_4 | 205 | 206 | *iOccG, inferior occipital gyrus* |
|  |  | LOcC _L(R)_2_1 | 207 | 208 | *msOccG, medial superior occipital gyrus* |
|  |  | LOcC _L(R)_2_2 | 209 | 210 | *lsOccG, lateral superior occipital gyrus* |
| **Subcortical Nuclei** | Amyg, Amygdala | Amyg_L(R)_2_1 | 211 | 212 | *mAmyg, medial amygdala* |
|  |  | Amyg_L(R)_2_2 | 213 | 214 | *lAmyg, lateral amygdala* |
|  | Hipp, Hippocampus | Hipp_L(R)_2_1 | 215 | 216 | *rHipp, rostral hippocampus* |
|  |  | Hipp_L(R)_2_2 | 217 | 218 | *cHipp, caudal hippocampus* |
|  | BG, Basal Ganglia | BG_L(R)_6_1 | 219 | 220 | *vCa, ventral caudate* |
|  |  | BG_L(R)_6_2 | 221 | 222 | *GP, globus pallidus* |
|  |  | BG_L(R)_6_3 | 223 | 224 | *NAC, nucleus accumbens* |
|  |  | BG_L(R)_6_4 | 225 | 226 | *vmPu, ventromedial putamen* |
|  |  | BG_L(R)_6_5 | 227 | 228 | *dCa, dorsal caudate* |
|  |  | BG_L(R)_6_6 | 229 | 230 | *dlPu, dorsolateral putamen* |
|  | Tha, Thalamus | Tha_L(R)_8_1 | 231 | 232 | *mPFtha, medial pre-frontal thalamus* |
|  |  | Tha_L(R)_8_2 | 233 | 234 | *mPMtha, pre-motor thalamus* |
|  |  | Tha_L(R)_8_3 | 235 | 236 | *Stha, sensory thalamus* |
|  |  | Tha_L(R)_8_4 | 237 | 238 | *rTtha, rostral temporal thalamus* |
|  |  | Tha_L(R)_8_5 | 239 | 240 | *PPtha, posterior parietal thalamus* |
|  |  | Tha_L(R)_8_6 | 241 | 242 | *Otha, occipital thalamus* |
|  |  | Tha_L(R)_8_7 | 243 | 244 | *cTtha, caudal temporal thalamus* |
|  |  | Tha_L(R)_8_8 | 245 | 246 | *lPFtha, lateral pre-frontal thalamus* |

**Table S4.** The detailed information on the ten brain regions without genetic information.

| **Lobe** | **Gyrus** | **Left and Right Hemisphere** | **Label ID** | **Anatomical and modified Cyto-architectonic descriptions** |
| --- | --- | --- | --- | --- |
| Frontal Lobe | SFG, Superior Frontal Gyrus | SFG_L(R)_7_4 | 8 | *A6dl, dorsolateral area 6* |
|  | MFG, Middle Frontal Gyrus | MFG_L(R)_7_5 | 24 | *A8vl, ventrolateral area 8* |
|  | IFG, Inferior Frontal Gyrus | IFG_L(R)_6_1 | 30 | *A44d, dorsal area 44* |
|  |  | IFG_L(R)_6_3 | 34 | *A45c, caudal area 45* |
|  |  | IFG_L(R)_6_5 | 38 | *A44op, opercular area 44* |
| Temporal Lobe | PhG, Parahippocampal Gyrus | PhG_L(R)_6_5 | 118 | *TI, area TI (temporal agranular insular cortex)* |
| Parietal Lobe | Parietal Lobule | SPL_L(R)_5_3 | 130 | *A5l, lateral area 5* |
|  | Parietal Lobule | SPL_L(R)_5_4 | 132 | *A7pc, postcentral area 7* |
|  | Pcun, Precuneus | PCun_L(R)_4_1 | 148 | *A7m, medial area 7(PEp)* |
| Occipital Lobe | LOcC, lateral Occipital Cortex | LOcC _L(R)_2_1 | 208 | *msOccG, medial superior occipital gyrus* |

**Table S5.** The detailed information of the 28 high-risk genes.

| **Gene** | **Entrez ID** | **Gene Description** | **Gene Family** |
| --- | --- | --- | --- |
| A2M | 2 | Alpha-2-Macroglobulin | extracellular matrix |
| ACE | 1636 | angiotensin I converting enzyme | metabolic enzyme |
| ACHE | 43 | acetylcholinesterase (Yt blood group) | metabolic enzyme |
| APBA1 | 320 | amyloid beta (A4) precursor protein-binding, family A, member 1 (X11) | signal transduction |
| APBB2 | 323 | amyloid beta (A4) precursor protein-binding, family B, member 2 (Fe65-like) | signal transduction |
| APLP1 | 333 | amyloid beta (A4) precursor-like protein 1 | other membrane protein |
| APLP2 | 334 | amyloid beta (A4) precursor-like protein 2 | other membrane protein |
| APOC1 | 341 | apolipoprotein C-I | transporter |
| APP | 351 | amyloid beta (A4) precursor protein (peptidase nexin-II, Alzheimer’s disease) | other membrane protein |
| BACE2 | 25825 | beta-site APP-cleaving enzyme 2 | other membrane protein |
| BCHE | 590 | butyrylcholinesterase | metabolic enzyme |
| BLMH | 642 | bleomycin hydrolase | metabolic enzyme |
| CASP3 | 836 | caspase 3, apoptosis-related cysteine peptidase | metabolic enzyme |
| CHRNA3 | 1136 | cholinergic receptor, nicotinic, alpha 3 | ion channel |
| CTSB | 1508 | cathepsin B | metabolic enzyme |
| DBN1 | 1627 | drebrin 1 | cytoskeletal protein |
| ESR1 | 2099 | estrogen receptor 1 | transcription factor |
| GSK3B | 2932 | glycogen synthase kinase 3 beta | kinase |
| IL1B | 3553 | interleukin 1, beta | peptide ligand |
| KCNIP3 | 30818 | Kv channel interacting protein 3, calsenilin | signal transduction |
| KLK6 | 5653 | kallikrein-related peptidase 6 | metabolic enzyme |
| LRP1 | 4035 | low density lipoprotein-related protein 1 (alpha-2-macroglobulin receptor) | other membrane protein |
| LRRC15 | 131578 | leucine rich repeat containing 15 | cell adhesion |
| MAPT | 4137 | microtubule-associated protein tau | cytoskeletal protein |
| PLAU | 5328 | plasminogen activator, urokinase | extracellular matrix |
| PSEN1 | 5663 | presenilin 1 (Alzheimer’s disease 3) other membrane protein | other membrane protein |
| PSEN2 | 5664 | 5664 presenilin 2 (Alzheimer’s disease 4) | other membrane protein |
| SORL1 | 6653 | sortilin-related receptor, L(DLR class) A repeats-containing | other membrane protein |

**Table S6.** The detailed information of the top 20 results for pathway and process enrichment analysis.

| **GO** | **Category** | **Description** | **P-value** |
| --- | --- | --- | --- |
| GO:0098662 | GO Biological Processes | inorganic cation transmembrane transport | 2.29087e-09 |
| GO:0007389 | GO Biological Processes | pattern specification process | 7.07946e-09 |
| GO:0099003 | GO Biological Processes | vesicle-mediated transport in synapse | 3.38844e-10 |
| R-HSA-9675108 | Reactome Gene Sets | Nervous system development | 3.0903e-08 |
| GO:0007420 | GO Biological Processes | brain development | 5.37032e-14 |
| GO:0099601 | GO Biological Processes | regulation of neurotransmitter receptor activity | 5.12861e-08 |
| GO:0034765 | GO Biological Processes | regulation of ion transmembrane transport | 3.80189e-12 |
| R-HSA-388396 | Reactome Gene Sets | GPCR downstream signalling | 5.88844e-09 |
| GO:0044057 | GO Biological Processes | regulation of system process | 1.90546e-10 |
| GO:0007268 | GO Biological Processes | chemical synaptic transmission | 7.24436e-16 |
| GO:1903530 | GO Biological Processes | regulation of secretion by cell | 1.25893e-08 |
| GO:0050803 | GO Biological Processes | regulation of synapse structure or activity | 1.8197e-08 |
| GO:0050806 | GO Biological Processes | positive regulation of synaptic transmission | 2.04174e-08 |
| GO:0007626 | GO Biological Processes | locomotory behavior | 1.14815e-08 |
| R-HSA-112316 | Reactome Gene Sets | Neuronal System | 4.46684e-17 |
| GO:0010975 | GO Biological Processes | regulation of neuron projection development | 6.16595e-08 |
| GO:0031175 | GO Biological Processes | neuron projection development | 9.33254e-18 |
| GO:0007610 | GO Biological Processes | behavior | 6.60693e-14 |
| GO:0050804 | GO Biological Processes | modulation of chemical synaptic transmission | 8.12831e-24 |
| WP4312 | WikiPathways | Rett syndrome causing genes | 8.70964e-08 |

**Table S7.** The percentage of variance explained by PLS components in AD vs. NC, MCI vs. NC, and significant brain regions in AD vs. NC.

|  | **AD vs. NC** | **MCI vs. NC** | **significant brain regions in AD vs. NC** |
| --- | --- | --- | --- |
| PLS1 | 16.84% | 13.86% | 26.76% |
| PLS2 | 11.09% | 12.89% | 14.20% |
| PLS3 | 8.35% | 6.28% | 7.50% |

**Table S8.** The overlapping subset of the related genes between the MCI and AD groups.

| Overlapped genes | | | | | | | |
| --- | --- | --- | --- | --- | --- | --- | --- |
| AASS | ACHE | AK3 | ANO2 | APOC1 | AQP1 | AQP5 | ATP11C |
| BNIP2 | BTN2A2 | C10or  f105 | CA12 | CAMK  MT | CCDC  191 | CCDC80 | CD226 |
| CD38 | CD44 | CD99 | CHODL | COLE  C12 | CREB  3L2 | CRNDE | CXCR4 |
| CYBA | CYBRD1 | CYP7B1 | DHRSX | DRD2 | ECEL1 | EFEMP1 | EFNA2 |
| EPHX2 | EYA1 | FABP6 | FAM167A-AS1 | FAM1  89A2 | FAM1  96A | FAM46A | FMO2 |
| FOXO1 | GBP3 | GINS3 | GPR149 | GPR183 | HADHB | HEBP1 | HES1 |
| HEY2 | HEYL | HPSE2 | HSDL2 | HVCN1 | ID4 | IL13RA1 | ITGB8 |
| KLHL13 | LAMA1 | LFNG | LINC0  0467 | LINC0  0886 | LINC0  1485 | LOC1019  27318 | MSN |
| MXRA8 | MYOM1 | NAV2 | NKAIN4 | NPC2 | NPL | NTSR2 | NUPR1 |
| OSBPL9 | PARD3B | PBX3 | PCBD2 | PDLIM5 | PIRT | PLEKHA4 | PLEKHA7 |
| PREX2 | RAB2B | RASL12 | RGS1 | RGS8 | RGS9 | RHOC | RMDN1 |
| RSPO4 | S100  PBP | SALL2 | SAMD11 | SCIN | SERP  INA3 | SERPINB1 | SINHCAF |
| SIX3 | SLITRK6 | SMIM10 | SMIM30 | SP110 | SPARC | SPATA13 | SRGAP1 |
| STK33 | STXBP4 | SUCLG2 | SULT1C4 | SUSD2 | SYND  IG1L | SYTL4 | TAL2 |
| TEX9 | THBS4 | TIMP3 | TNC | TTC38 | UACA | VAT1 | WDR49 |
| WWTR1 | ZFHX3 | ZFHX4 | ZFP36L1 | ZFP36L2 | ZIC1 | ZIC3 | ZIC4 |

**Table S9.** Demographics of six adult donors in the AHBA dataset.

| **Donor** | **Number of cohorts** | **Age** | **Sex** | **Ethnicity** | **Post-mortem interval** |
| --- | --- | --- | --- | --- | --- |
| H0351.2001b | 946 | 24 | Male | African American | 23h |
| H0351.2002 | 893 | 39 | Male | African American | 10h |
| H0351.1009 | 363 | 57 | Male | Caucasian | 25.5h |
| H0351.1012 | 529 | 31 | Male | Caucasian | 17.5h |
| H0351.1015 | 470 | 49 | Female | Hispanic | 30h |
| H0351.1016 | 501 | 55 | Male | Caucasian | 18h |


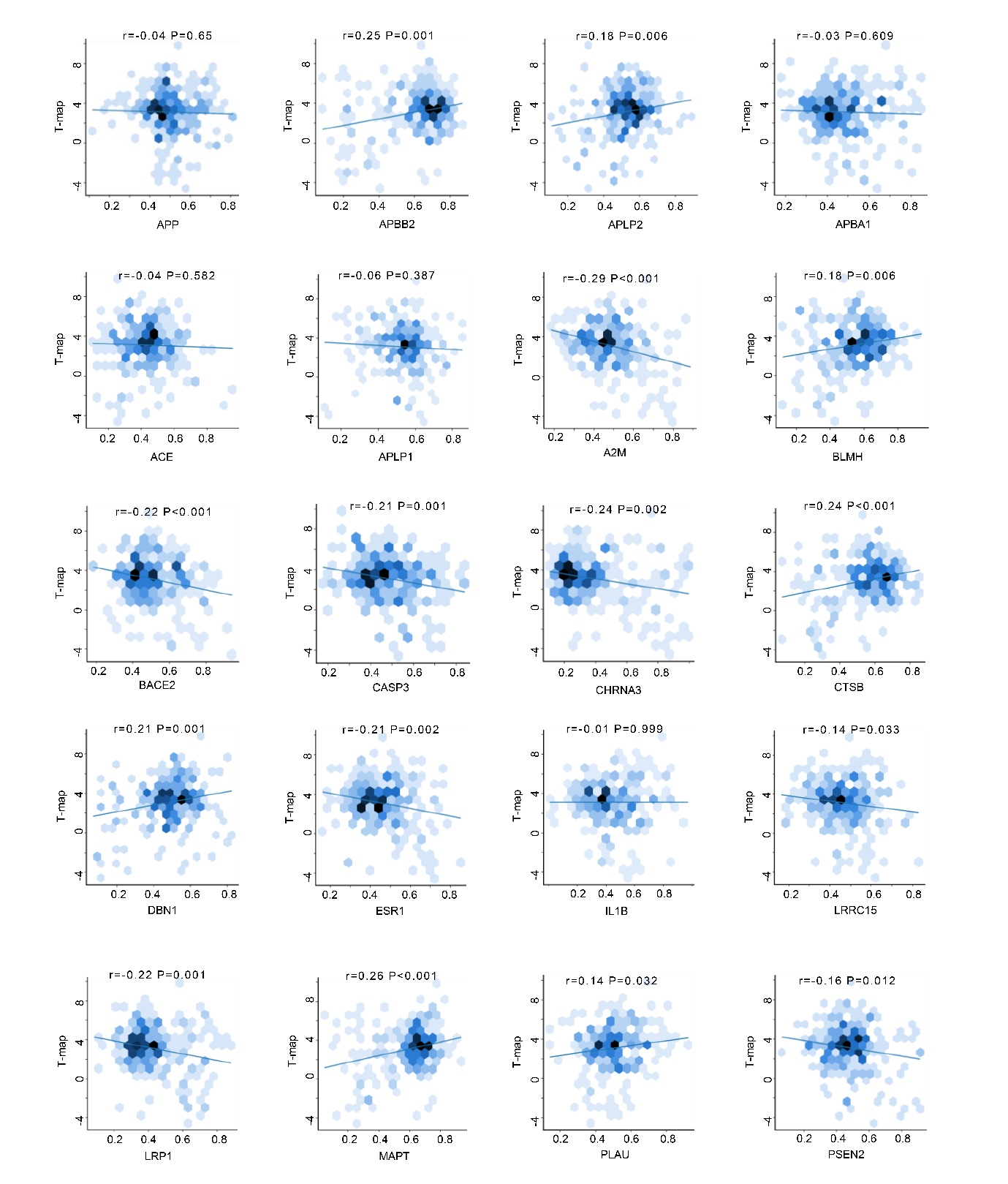


**Figure S1.** The correlation between the other twenty AD-related genes and the T-map of the RMCS in AD vs. NC.

**
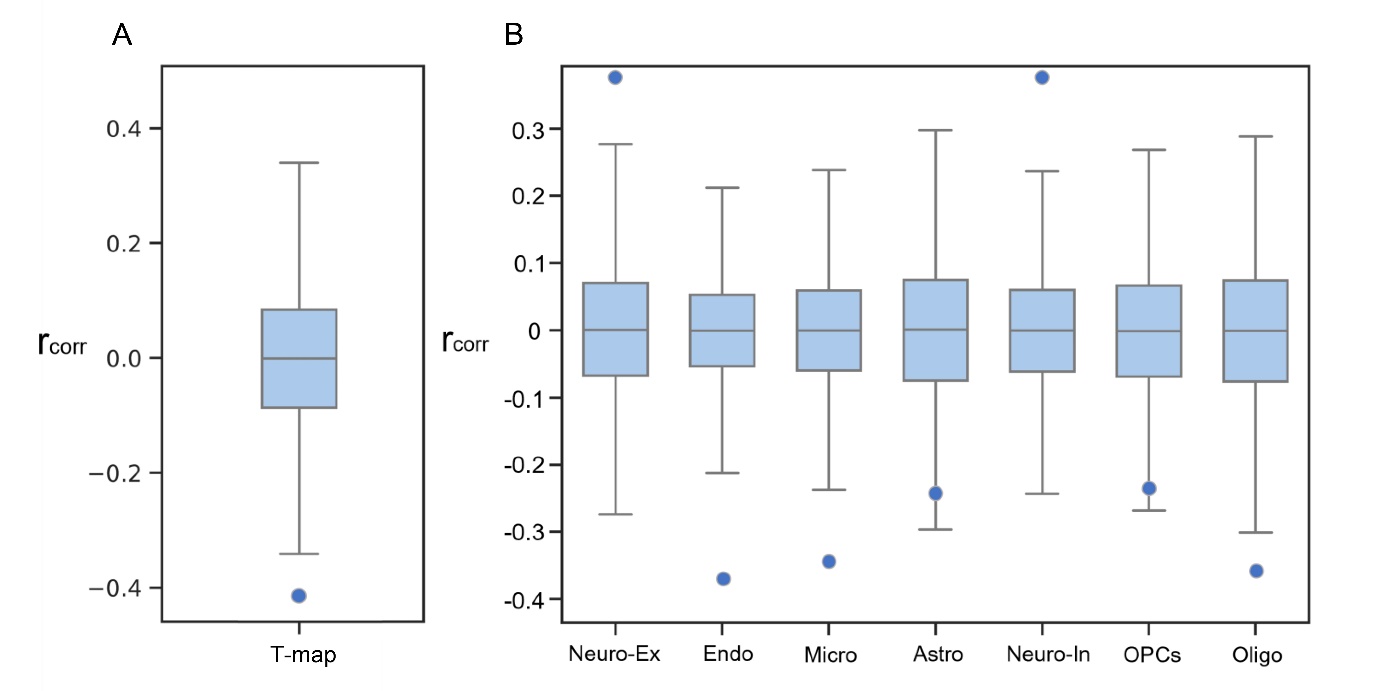
**

**Figure S2.** The r values which were obtained with variogram-based spatially auto-correlated null distributions of gene expression compared to the (A) T-map of the RMCS in AD vs. NC and (B) cell types.


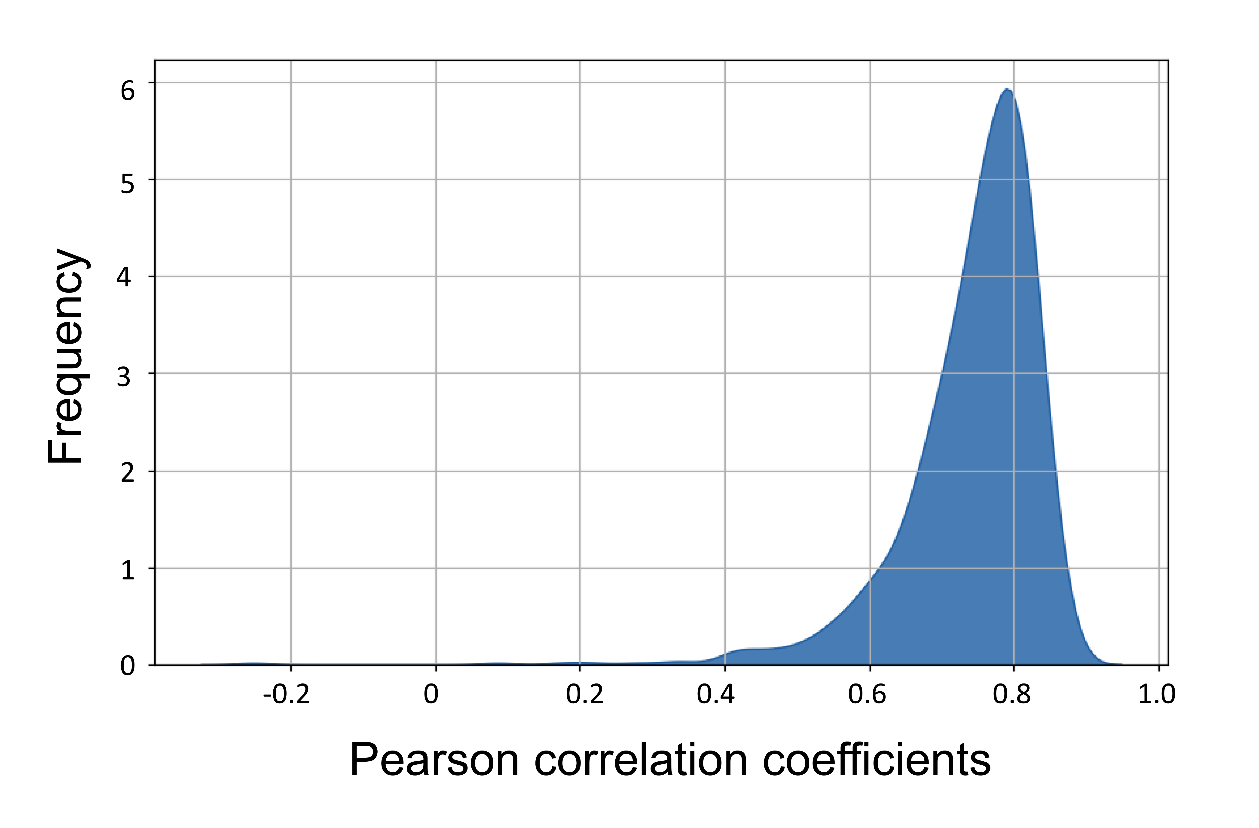


**Figure S3.** The distribution of Pearson correlation coefficients between the statistical significance resulting of random selections and the outcomes of our initial ANOVA analyses.**
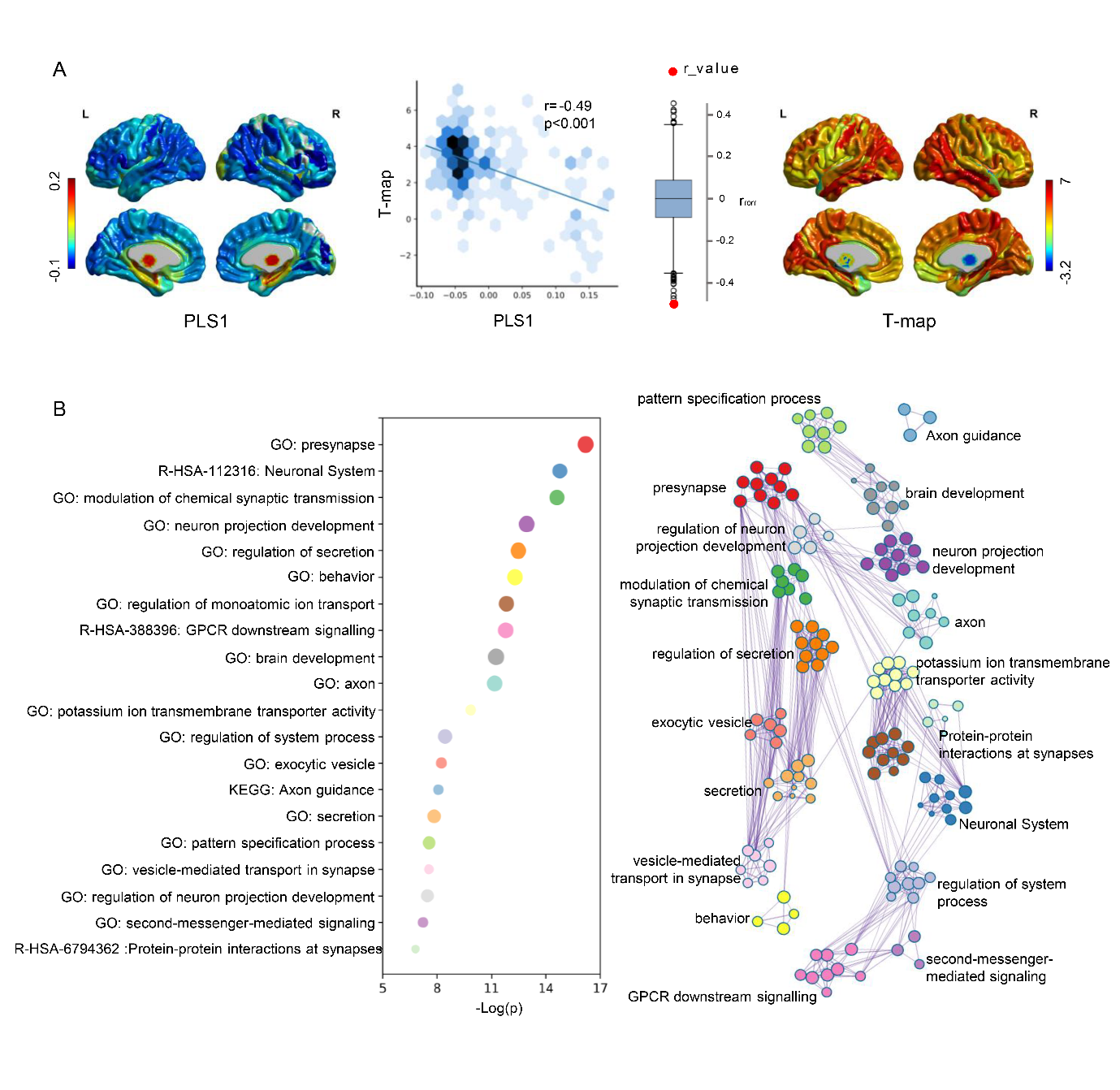
**

**Figure S4.** Functional enrichment of gene transcripts between the difference MCI and NC. (A) The correlation between the PLS1 score and the T-map of the RMCS in MCI vs. NC and r value was obtained with variogram-based spatially auto-correlated null distributions. (B) The results of GO term and Reactome pathway. The size of the node is proportional to the number of input genes contained in the term, and its color represents cluster identity.


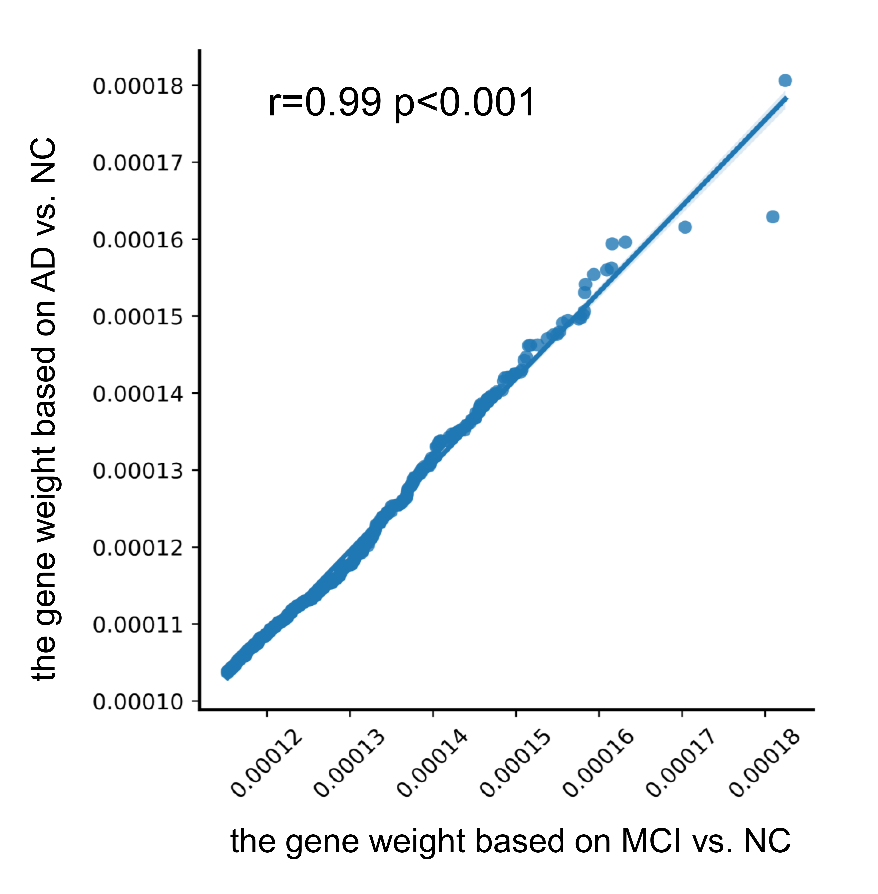


**Figure S5.** The correlation between the weights of enriched genes in AD and MCI.


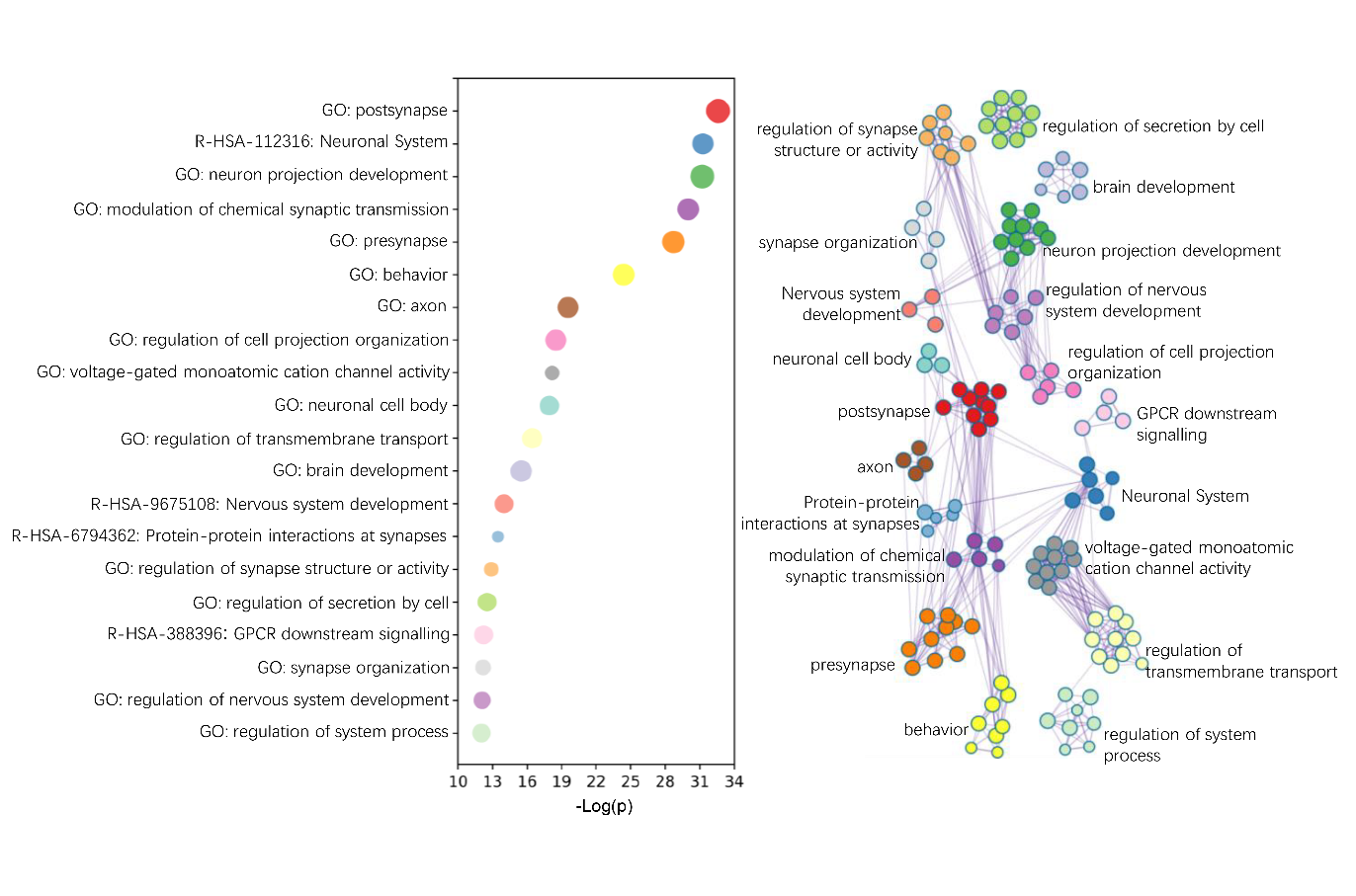


**Figure S6.** Functional enrichment results of the top 1000 genes. The size of the node is proportional to the number of input genes contained in the term, and its color represents cluster identity.


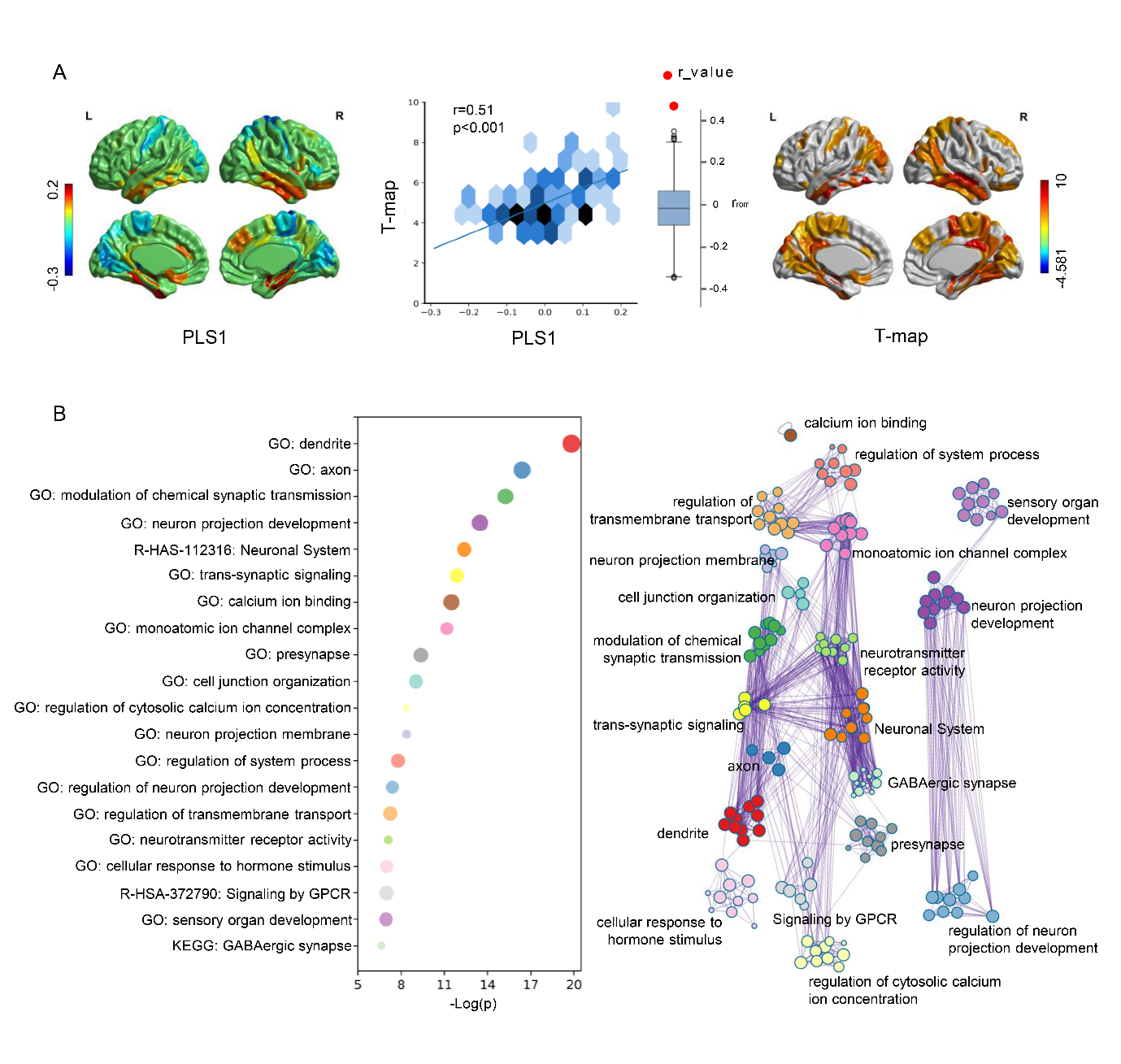


**Figure S7.** Functional enrichment of gene transcripts of the significant brain regions between AD and NC. (A) The correlation between the PLS1 score and the T-map of the RMCS in significant brain regions and r value was obtained with variogram-based spatially auto-correlated null distributions. (B) The results of GO term and Reactome pathway. The size of the node is proportional to the number of input genes contained in the term, and its color represents cluster identity.


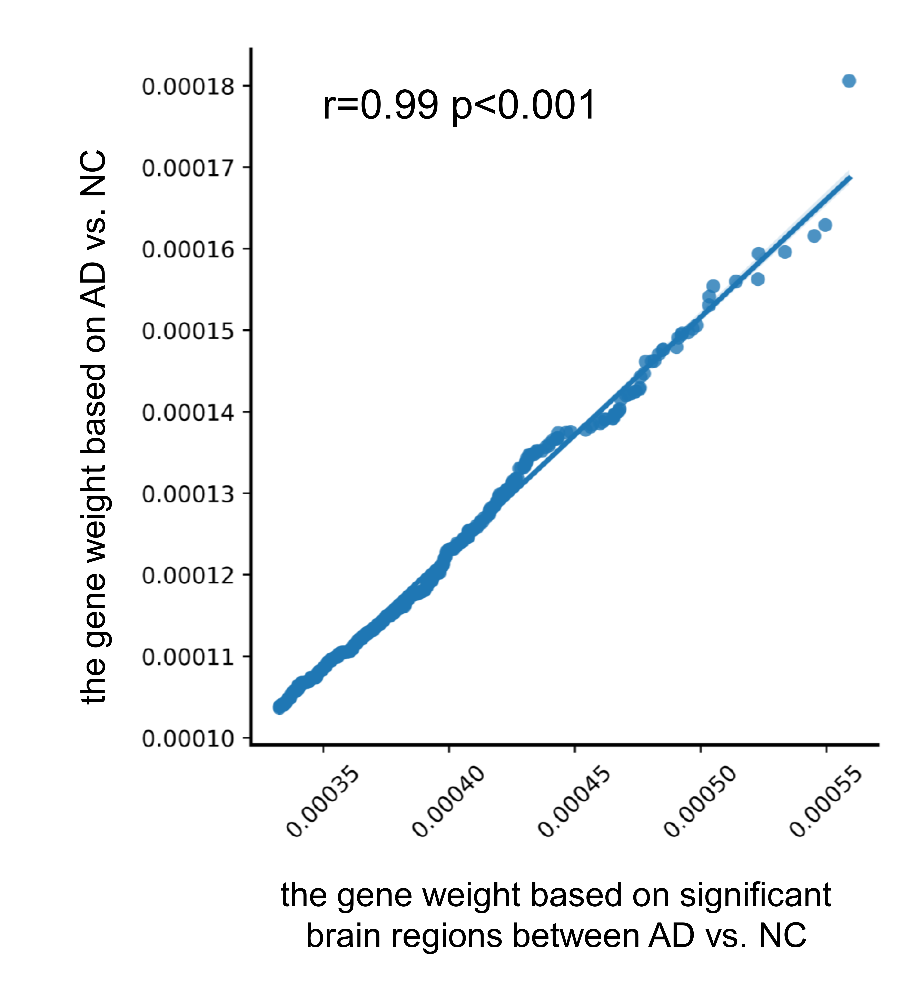


**Figure S8.** The correlation between the weights of enriched genes in AD and significant brain regions in AD.

Fan, L., Li, H., Zhuo, J., Zhang, Y., Wang, J., Chen, L., . . . Laird, A. R. (2016). The human brainnetome atlas: a new brain atlas based on connectional architecture. *Cerebral CORTEX, 26*(8), 3508-3526.

Zhao, K., Zheng, Q., Che, T., Dyrba, M., Li, Q., Ding, Y., . . . Li, S. (2021). Regional radiomics similarity networks (R2SNs) in the human brain: Reproducibility, small-world properties and a biological basis. *Netw Neurosci, 5*(3), 783-797. doi:10.1162/netn_a_00200
